# Selection on metabolic pathway function in the presence of mutation-selection-drift balance leads to rate-limiting steps that are not evolutionarily stable

**DOI:** 10.1186/s13062-016-0133-6

**Published:** 2016-07-08

**Authors:** Alena Orlenko, Ashley I. Teufel, Peter B. Chi, David A. Liberles

**Affiliations:** Center for Computational Genetics and Genomics and Department of Biology, Temple University, Bio-Life Building, 1900 N. 12th Street, Philadelphia, PA 19122-1801 USA; Department of Molecular Biology, University of Wyoming, Laramie, WY 82071 USA; Department of Mathematics and Computer Science, Ursinus College, Collegeville, PA 19426 USA

**Keywords:** Metabolic pathway evolution, Systems biology, Population genetics

## Abstract

**Background:**

While commonly assumed in the biochemistry community that the control of metabolic pathways is thought to be critical to cellular function, it is unclear if metabolic pathways generally have evolutionarily stable rate limiting (flux controlling) steps.

**Results:**

A set of evolutionary simulations using a kinetic model of a metabolic pathway was performed under different conditions to evaluate the evolutionary stability of rate limiting steps. Simulations used combinations of selection for steady state flux, selection against the cost of molecular biosynthesis, and selection against the accumulation of high concentrations of a deleterious intermediate. Two mutational regimes were used, one with mutations that on average were neutral to molecular phenotype and a second with a preponderance of activity-destroying mutations. The evolutionary stability of rate limiting steps was low in all simulations with non-neutral mutational processes. Clustering of parameter co-evolution showed divergent inter-molecular evolutionary patterns under different evolutionary regimes.

**Conclusions:**

This study provides a null model for pathway evolution when compensatory processes dominate with potential applications to predicting pathway functional change. This result also suggests a possible mechanism in which studies in statistical genetics that aim to associate a genotype to a phenotype assuming independent action of variants may be mis-specified through a mis-characterization of the link between individual gene function and pathway function. A better understanding of the genotype-phenotype map has potential applications in differentiating between compensatory changes and directional selection on pathways as well as detecting SNPs and fixed differences that might have phenotypic effects.

**Reviewers:**

This article was reviewed by Arne Elofsson, David Ardell, and Shamil Sunyaev.

**Electronic supplementary material:**

The online version of this article (doi:10.1186/s13062-016-0133-6) contains supplementary material, which is available to authorized users.

## Background

A long standing goal in molecular evolution and comparative genomics is to understand how genes and their functions evolve. Molecular evolutionary and statistical genetics analyses have commonly treated protein function independently of the functions of other proteins and without consideration of genotype-phenotype maps. However, mutation works at the level of the gene, while selection works at the level of the organism in a population in an ecosystem. One critical component of the interplay between molecular biology and organismal biology is the metabolic pathway that combines the actions of multiple proteins (enzymes) in the generation of energy and molecular building blocks. Systems of differential equations based upon Michaelis-Menten kinetics have become a common modeling tool for describing the function of metabolic pathways [1].

But how do pathways evolve and how do their constituent members co-evolve? Within a given pathway, various enzymes catalyze reactions at different efficiencies and rates. Rate-limiting steps are the bottlenecks in biochemical pathways and can serve as important points of regulation. Kacser and Burns [2] established the concept of flux control enzymes in a pathway, with a given set of enzyme rate constants. The distribution of rate-limiting steps varies across different biochemical networks, although current biochemical thought from network control theory is that selection has a strong role in maintaining efficient pathway function and regulation through the most controllable points [3–15]. Specific examples in glycolysis are given in [16]. A correlate of this is an expectation of evolutionary stability of rate limiting steps when there is negative (stabilizing) selection on pathway function (for example, steady state flux that is not selected to change) [5]. This issue, however, has not been seriously addressed in the biochemical literature. Related biochemical expectations suggest that the observed distribution of rate-limiting steps is driven by pathway architecture [4, 12, 14]. An examination of the BioModel Database [17] showed glycolysis as the only pathway with data from multiple species and it shows no evidence for conserved rate limiting steps controlling steady state flux [16].

Further, it is unclear from a population genetic perspective that stabilizing selection on steady state flux should give rise to evolutionarily stable rate limiting steps in the presence of mutation-selection balance (see [18, 19] for other studies on the role of mutation-selection balance on molecular systems). The effect of new mutations on fitness has been characterized [20] and has large fractions of both strongly deleterious (lethal) mutations and slightly deleterious mutations. The frequency and magnitude of slightly deleterious mutations depends upon the mutational space surrounding the protein sequence and is linked to its stability and activity [21, 22]. At the two ends of the spectrum, the globally most active sequence will have only degenerative changes possible, while the globally least active sequence will have only activating changes possible. In between, the proportion of changes that increase or decrease activity will depend upon the current activity. With such a mutational process acting on molecular phenotypes (rather than fitnesses directly), it is expected that enzymes with excess activity will accumulate changes that reduce their activity until they affect pathway flux and are acted upon by selection. At the level of an individual enzyme within a pathway, these dynamics have been described [23].

**Fig. 1:**
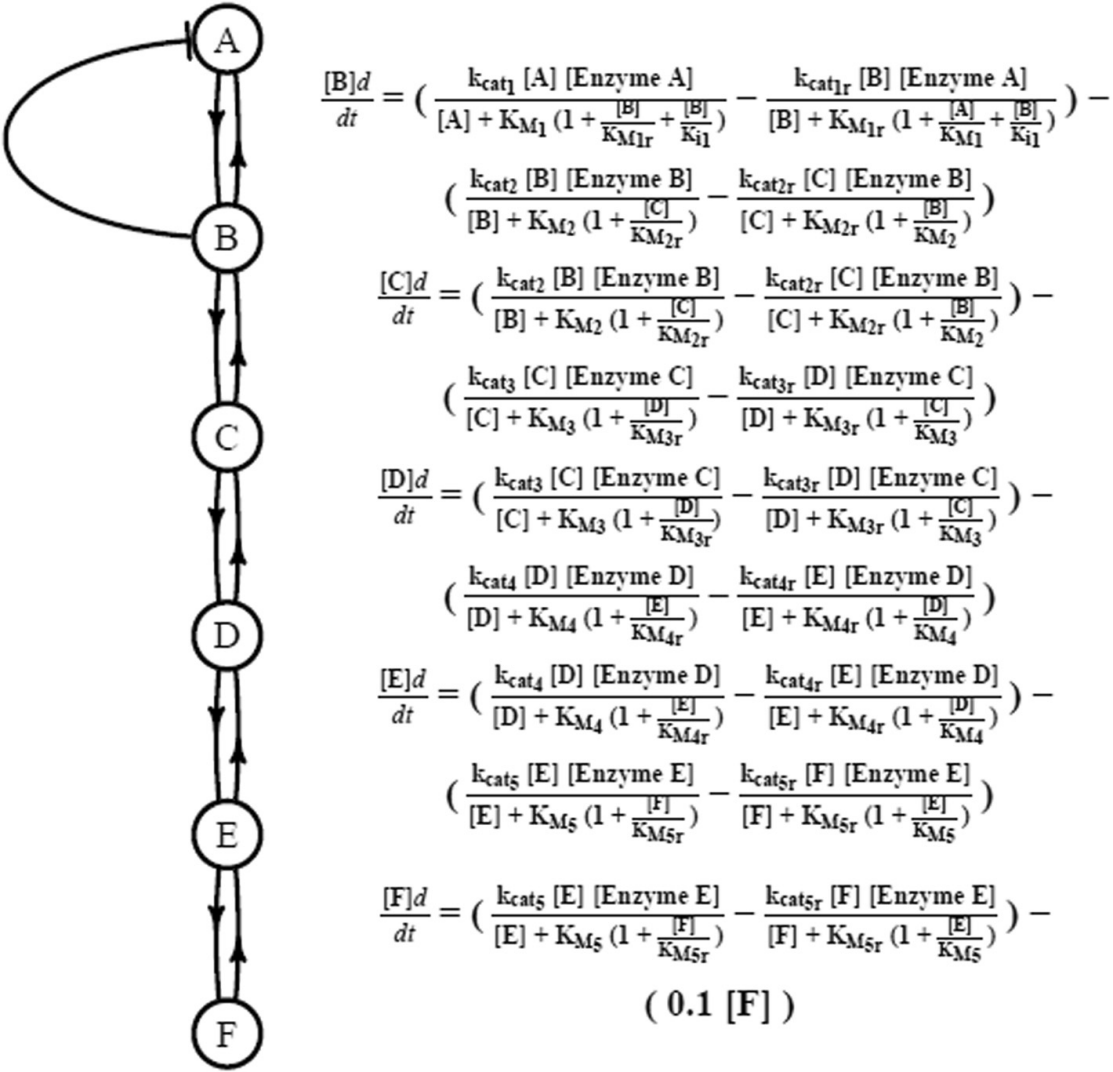
The simplified pathway that was simulated is shown. This pathway contains features of both glycolysis [35] and the methylglyoxal pathway [36]. A constant concentration of compound A is converted to compound F and the steady state flux is measured

In this study we use simulations on a simplified pathway (Fig. 1) to examine the nature of mutation-selection-drift balance and enzyme co-evolution, towards an understanding of the evolutionary stability of rate limiting steps. In addition to selection on flux, two additional biological considerations that have been suggested in the literature are included, selection against the cost of mRNA and protein synthesis to prevent wasteful expression [24] and selection against the accumulation of high concentrations of intermediate compounds that may be toxic to a cell [13]. We have also tested the role of effective population size and of biophysical assumptions on the number of mutational degrees of freedom on the evolutionary dynamics. The analysis suggests that rate limiting steps may not be stable over long evolutionary periods.

## Results

### Mutation-selection-drift balance and rate limiting steps

Pathway evolution was simulated according to several sets of mutational processes and selective regimes to evaluate the evolutionary dynamics with simultaneous mutational and selective pressures acting on pathway function. After 20,000 generations, all experiments except the mutation-only negative control (where selection was absent) showed that the fitness equilibrium had been reached (Additional file 1: Figures S20-S21). However, when the mutational process was designed to mimic biological mechanisms and adaptive mutation was limiting, there was still co-evolutionary directional movement in some parameters without fitness effects, most notably K_M_ (Additional file 1: Figures S5-S19).

**Fig. 2:**
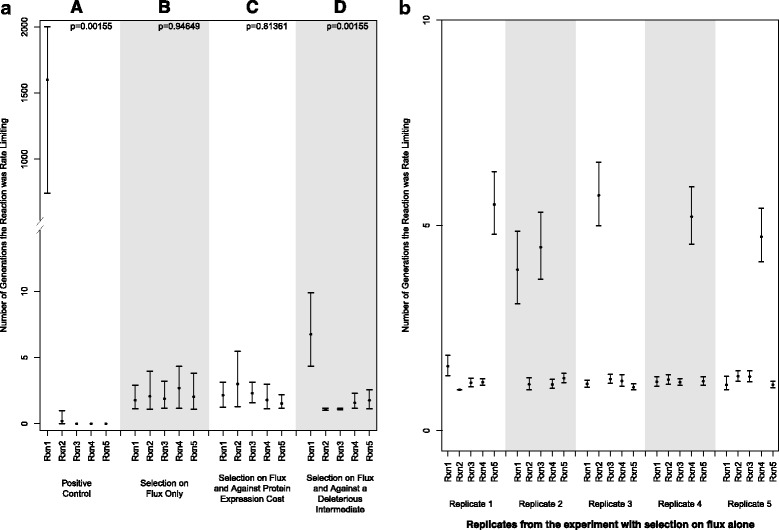
**a** The evolutionary stability of rate limiting steps is shown through the average number of generations each step was found to remain rate limiting, once it emerged as rate limiting. Error bars delineate 95 % bootstrap confidence intervals for each set of experimental conditions, and p-values are for the null hypothesis that the average is constant across each reaction within the experiment. **b** The variability among replicates within the experiment investigating selection on flux only is shown. Error bars delineate 95 % bootstrap confidence intervals

When simulating the evolution of metabolic pathways in a forward population genetic regime and consistent with previous findings [9, 14], a neutral (towards molecular phenotype) mutational process led to evolutionarily stable rate limiting steps, particularly when specific intermediates were selected as deleterious at high concentration (Fig. 2a, panel A) [13]. However, when mutational pressure was applied according to an expected distribution acting upon molecular phenotypes, mutation-selection balance emerged and no step was stable for a longer evolutionary period than other steps (Fig. 2a, panel B). All steps spent only brief evolutionary periods as rate limiting.

When an intermediate occurs at high concentration generating concentration-dependent toxicity (methylglyoxal as an example) or becomes subject to cross-reactivity with other pathways and enzymes that bind at lower K_M_ to such substrates, this can create a selective pressure against a high concentration of the intermediate. When a selective pressure was applied against a particular intermediate (intermediate B), an increase in the evolutionary period that the producing enzyme was rate limiting was observed (Fig. 2a, panel D). However, these experiments still did not display rate-limiting steps with long evolutionary stability and mutation-selection balance dominated the evolutionary dynamics. The increase (of about 5 generations on average) in the half-life of the first reaction as a rate-limiting step may be due to selection against overly active enzyme A when compensatory changes to enzyme B are limiting. Enzyme B did not show significant differences in the time spent rate limiting when compared with the other 3 enzymes. The overall proportion of time that each reaction spent as rate-limiting is shown in Table 1. In this instance, there was an increase in the period of time that the reaction leading to the production of the deleterious intermediate was rate-limiting, suggesting that sampling of genomes would observe that this step is flux controlling most frequently, even though it is not evolutionarily stable as flux controlling.

**Table 1:** The overall proportion of generations that each reaction spent as rate limiting is shown, pooled across each replication, for each experiment

	Reaction 1	Reaction 2	Reaction 3	Reaction 4	Reaction 5
Positive control	0.9998	0.0002	0.0000	0.0000	0.0000
Selection on flux only	0.1201	0.2048	0.1499	0.3314	0.1937
Selection on flux and against protein expression cost	0.2137	0.2732	0.1961	0.1790	0.1380
Selection on flux and against a deleterious intermediate	0.7018	0.0259	0.0421	0.0814	0.1488

It is important to note that within each experiment, there was variability among the replicates. That is, within each replicate, a particular reaction may have had higher numbers of consecutive generations in which it was rate limiting, and this was not necessarily constant among all replicates. In particular, for the experiment shown in Fig. 2a, panel B, the variability among replicates is shown in Fig. 2b.

**Fig. 3:**
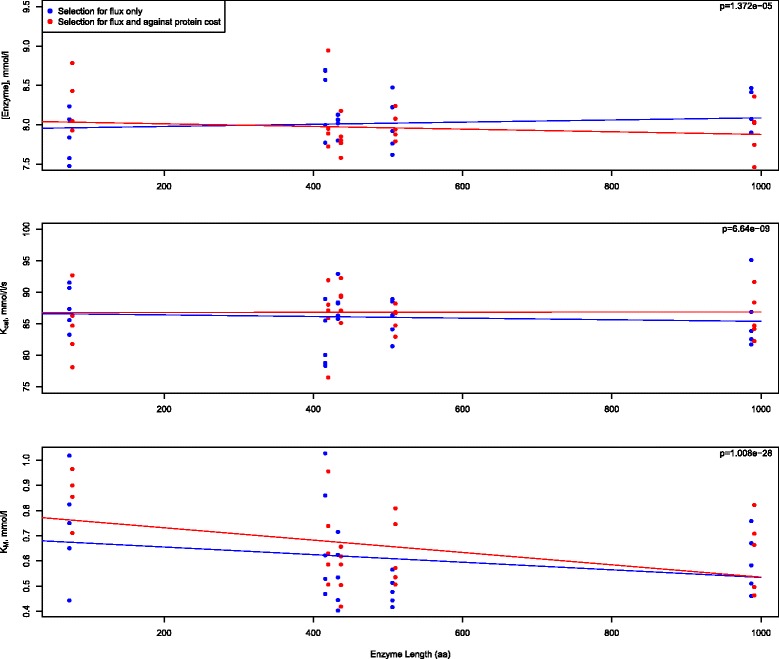
In evaluating the influence of selection against the cost of protein expression on the evolutionary dynamics or parameters, the relationship of enzyme length to **a** enzyme concentration, **b** k_cat_, and **c**, and K_M_ is shown when selection acts on expression cost in addition to flux and when it acts only on flux. Each point represents the average value from each replicate. In each panel, p-values correspond to the question of whether the slopes are different from each other, as assessed by mixed-effects interaction models

Another important mechanism that has been discussed is that of selection against expression cost [24]. When the expression cost is included in the fitness function (Fig. 2a, panel C), there is no difference between the enzymes in the period found to be rate limiting. However, there are trade-offs in the individual parameters induced by this selective pressure (Fig. 3). While selection against flux alone shows no position-specific effects in enzyme concentration, the relationship between enzyme length (expression cost) and flux shows a negative slope (Fig. 3a). The expected patterns are also observed with k_cat_ (Fig. 3b; positive slope) and with K_M_ (Fig. 3c; more negative slope). That the K_M_ values do not fully compensate for the expression, especially in the smallest enzymes may be indicative of a combination of weak selection and a waiting time for beneficial mutations that are governed by a more complex landscape with the added expression cost term.

### Allele segregation within population

In order to assess the consistency of the observations made in this simulation with population genetic expectations given the mutational profile, the amount of segregating variation in the population was characterized. As estimated from Kimura and Crow [25] as described in the Methods section, assuming neutrality, the expected number of alleles for each parameter in the population is 1.6 alleles per parameter segregating at any time. The observed values that were calculated from the selection on flux only experiment are greater than the expectation when neutral, but of the same order of magnitude. Over 2000 generations, the mean for all of the parameters was found to be 2.75 with a standard deviation of 0.33. The minimum and the maximum of the range were found to be 1.85 and 3.77 correspondingly. For the reaction parameters involved only in the forward direction, the mean for 2000 generations was found to be 2.48 (standard deviation 0.37), with the range minimum of 1.53 and maximum of 3.8. The difference in the parameters reflects the action of selection, particularly with regard to the reverse parameters.

### Patterns of co-evolution

**Fig. 4:**
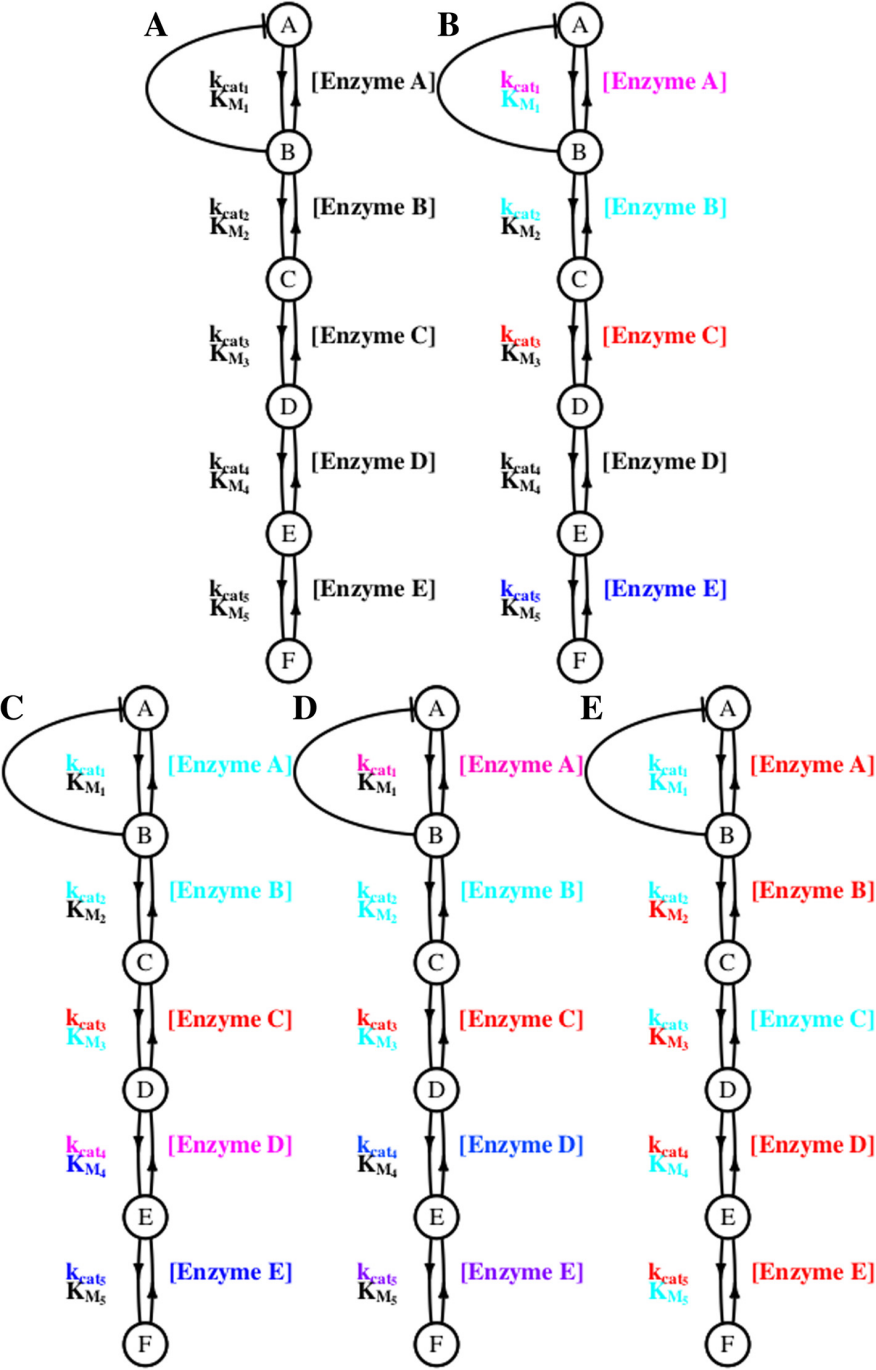
Clustering of co-evolving parameters across simulations. Colors represent parameters which are found to cluster together (at the 5 % level), while black parameters were not found to have significant associations with clusters. Panels show results from simulations with **a** mutation only, **b** selection on flux only, **c** selection on flux and against total expression cost, **d** selection on flux and against a high concentration of a deleterious intermediate, and **e** non-biological neutral mutation and selection on flux and for the first reaction to be rate limiting

When mutation-selection-drift balance occurs under negative (stabilizing) selection, individual parameter values are still changing. After controlling for systematic directional change in K_M_, patterns of parameter co-evolution can be examined as a characteristic of the co-evolutionary fitness landscape. As expected, without selection, mutational pressure alone results in no significant clusters (as assessed via bootstrapping) (Fig. 4a). When selection acted on flux alone (Fig. 4b) and when it acted on both flux and against a high concentration of a deleterious intermediate (Fig. 4d), the parameters of an enzyme formed significant clusters, largely independent for each enzyme. When selection acted on flux and against total expression cost (Fig. 4c), clusters surprisingly corresponded to positions within a pathway rather than to enzyme length. This may be due to the complex fitness landscape in this simulation that was limited by adaptive mutation, although the exact cause of this particular pattern is not immediately clear. Selection for the first step to be rate-limiting with a neutral mutational process (Fig. 4e) also showed fewer clusters with k_cat_ values and K_M_ values clustering by pathway position, consistent with prior observations [9].

### Is mutation-selection-drift balance just drift in a small population in disguise?

**Fig. 5:**
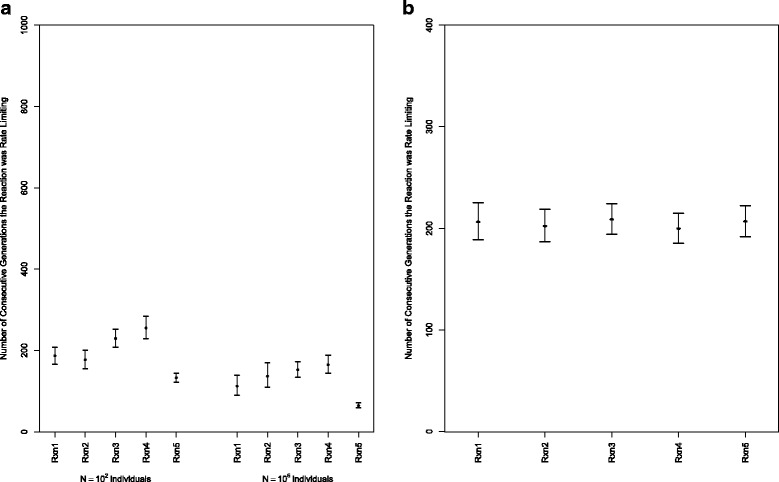
**a** The evolutionary stability of rate-limiting steps for experiments with small (10^2^) and large (10^6^) population sizes was evaluated, with altered simulation assumptions from Fig. 2. Error bars delineate 95 % bootstrap confidence intervals found across 30 replicates for both sets of population size. **b** The evolutionary stability of rate-limiting steps, subject to the constraint dictated by Haldane’s relationship, is shown. Error bars delineate 95 % bootstrap confidence intervals found across 30 replicates

It might be conceived that the results shown in Fig. 2 are an artifact of a small effective population size and simply reflect drift, arguing that any semblance of mutation-selection balance as a limit to pathway control will disappear with a larger effective population size. To test this, a new simulation scheme was developed without explicit individuals, but maintaining explicit generations. This approximation to the population genetic process (with added caveats as described in Methods) enabled us to evaluate the average length of time a step remained rate limiting under an identical to above small (10^2^) population size and under a much larger population size (10^6^) with selection just on metabolic flux. As seen in Fig. 5a, a very similar pattern of the distribution of rate limiting steps is obtained with the large and small population sizes, suggesting that mutation-selection balance operates in both small and large population sizes on metabolic pathways, consistent with our expectations. The scaling of the number of generations that differs between Figs. 2 and 5 is a product of the different mutational and fixation processes in the different experiments and the presence of segregating variation from a high mutation rate in the first set of experiments. In that case, shifts were due to population dynamics rather than the fixation of new mutations.

### Haldane’s relationship and mutational processes

Haldane’s relationship describes the relationship between the various kinetic parameters in establishing an equilibrium that is consistent with thermodynamic observations of energy differences between reactants and products. In the simulations shown thus far, the mutational parameters are independently free to vary. Haldane’s relationship constrains the values of the parameters as acted upon by mutation by the thermodynamics of the reaction, although the joint effects of mutations are not currently modelable (see [16] for some discussion of this in the context of glycolysis as well as [26]). In the simplest case, Haldane’s relationship reduces 4 parameters to 3° of freedom, although more degrees of freedom are added with additional products and substrates, regulation, cofactors, and more complex equilibria. It is well known that modulation of K_M_ is used biologically to regulate the direction of the reaction [26]. However, the precise scheme that was simulated under had one too many degrees of freedom, so the effect of this was tested.

In the experiment constrained by Haldane’s relationship, the evolutionary stability of the rate-limiting steps has a similar average generation length of each reaction as rate-limiting, which is approximately 200 generations in both cases (Fig. 5a, b). Simulating under Haldane’s relationship generates a noticeably more flat distribution across reactions steps as well as less variance.

## Discussion

Low evolutionary stability of rate-limiting steps caused by mutation-selection balance appears to be an expectation for control in metabolic pathways when pathway flux is under negative (stabilizing selection), even when there is a preference for a particular reaction to be flux controlling (as in the reaction leading to a deleterious intermediate). It is clear that in nature, not all pathways are under selection for a constant flux, but may be temporally regulated. More complex regulatory schemes are expected to result in a more complex landscape and longer times to reach a fitness equilibrium, but mutation-selection-drift balance should still play an important role. Relatedly, processes that constantly shift the fitness equilibrium, such as shifting selection (see for example [27]) or shifting population sizes might be expected to show interesting evolutionary dynamics. Although the fitness equilibrium is never reached, this would provide a very different mechanism of generating flux controlling steps than control theory suggests.

In this study, only a linear pathway was examined. There is no reason to expect mutation-selection balance to not apply to branched pathways or cycles, although the dynamics of equilibration may in fact be different. This remains an interesting topic for future study.

A further layer of complexity is that this work has proceeded with a fixed network whereas network structure evolves in natural systems. Duplication [28] and the existence and evolution of promiscuous functions [29, 30] are known to give rise to specific processes of network growth [31]. The dynamics of this type of differential equation system evolution have been studied in a community ecology setting [32] and the co-evolutionary landscapes that emerge may be different from those with a static structure.

With an understanding of the co-evolution of parameters under negative selection, it will be interesting to observe if this pattern changes when positive directional selection is applied to a pathway flux. This would give a probabilistic basis to examining patterns of co-evolution in a pathway to differentiate between compensatory processes and directional selection. These models could also potentially be used to differentiate between negative and positive directional selection in an Approximate Bayesian Computing framework, where constraint on pathway flux gives rise to lineage-specific patterns of enzyme evolution that can be compared to data from gene family analysis.

Lastly, one debate that has consistently arisen in the molecular evolution community is that of the relative importance of changes in gene expression and changes in coding sequence evolution [33]. Mechanistic frameworks like this with roots in either a Boltzmann Distribution or Michaelis-Menten Kinetics, when coupled to a protein level mutational model (see for example [34]), have the potential to describe the mutational opportunity to affect phenotypes through changes in either protein concentrations or protein coding sequence function parameters (like K_M_ or k_cat_ although predictions on enzymatic reactions are more complex than binding). Deviations from this mutational opportunity (for example, from additional levels of constraint) would be informative about the molecular nature of both compensatory and adaptive evolution.

Relatedly, the field of statistical genetics has commonly made an assumption that the action of a variant is constant against all genetic backgrounds. In the simulations here, the effect of a variant that reduces enzyme activity will have a flux and fitness effect in some parameter (genetic) backgrounds and not in others. Statistically, this averaging would result in low power to detect causal variants. An understanding of the dynamics associated with processes like mutation-selection balance could be used to generally improve models used for understanding the genotype-phenotype map in various biological systems, including in human genetic disease.

## Conclusions

Many studies in comparative genomics study each gene in isolation and thereby miss the equilibrium that mutation, selection, and drift generate, including inter-molecular compensatory changes. Under several population genetic and selective regimes, the dynamics of enzyme co-evolution with ultimate negative selection on pathway flux were characterized, resulting in a general lack of evolutionarily stable rate-limiting steps. From this, expected patterns of enzyme co-evolution with negative selection were generated using a clustering approach. This ultimately provides a null model for pathway evolution under stabilizing selection.

## Methods

### Simulated evolution of metabolic pathways

To evaluate the role of mutation-selection-drift balance in biochemical pathway evolution, a population of cells with a key metabolic pathway was evolved under different selective schemes. The simplified kinetic model designed to capture features of glycolysis [35] and methylglyoxal metabolism [36] is shown in Fig. 1. The glycolysis-like aspects of the pathway include the feedback loop (as an approximation to glycolysis regulation) and the synthesis of final metabolite F as analogous to pyruvate in a linear pathway. The methylglyxoxal-like pathway elements include the toxic intermediate (B) as analogous to methylglyoxal (a highly toxic intermediate) and again, the synthesis of the final metabolite (F) is analogous to pyruvate.

This model is expressed in terms of a system of ordinary differential equations where reactions are described by reversible Michaelis-Menten kinetics. Each enzyme has parameters for enzyme concentration [Enzyme] (mmol/l), the catalytic constant (k_cat_) (mmol/l/s), the Michaelis constant for the substrate (K_M_) (mmol/l), the reversible catalytic constant (k_catr_) (mmol/l/s), and the Michaelis constant for the product (K_Mr_) (mmol/l). The kinetic model has a single inhibitory reaction that is described in the system by the inhibition constant K_I_ (mmol/l). The COPASI [37] modeling environment is used to solve this system of equations. The steady state solution is used, with a constantly replenishing concentration of A and mass action to utilize F, as described in Additional file 1: Table S1.

In order to model the evolutionary process, a forward time simulation with discrete generations is employed. In general, the simulation represents each individual in the population as an instance of the described model, subjecting this model individual to mutations which may elicit fitness effects, and then sampling individuals based on fitness to populate the next generation using weighted sampling with replacement. The pathway architecture remains unchanged during the course of the simulations. These simulations proceed by establishing an initial population of 100 homogenous individuals with parameter values given in Additional file 1: Table S1. Because a set of differential equations must be solved for each individual in each generation, the population size was limited by computational capacity. It is not expected that the results obtained in this study are driven by the size of the population. Each forward simulation was repeated 5 times. Mutations were introduced with a probability of 3*10^−3^ per parameter per individual per generation. K_M_ and k_cat_ were treated as evolving independently, although there is a mechanistically unpredictable degree of dependence (and link to protein stability) in their evolution in nature from current understanding, as described below. The mutational effect on the catalytic rate constant and enzyme concentration (both indicated by p below) are derived from a normal distribution with variable mean ${\mu}_{n_1}$, where

$$ {\mu}_{n_1}=-0.01{e}^{c*\cdot {p}_{n_1-1}} $$

The mutational effects on the binding constants (K) are described by a standard normal distribution with a variable mean ${\mu}_{n_2}$,

$$ {\mu}_{n_2}=\frac{1}{-0.01{e}^{c\cdot {K}_{n_2-1}}} $$

The index value c is used to scale the mutational effects, with the following values for each constant:

$$ c=\left\{\begin{array}{c}\hfill 2.5\times {10}^{-2},\wedge enzymeconcentration\hfill \\ {}\hfill 2.5\times {10}^{-2},\wedge inhibitionconstant\hfill \\ {}\hfill 1.0\times {10}^{-2},\wedge catalyticconstant\hfill \\ {}\hfill 3.{3}^{\prime}\times {10}^{-4},\wedge reversiblecatalyticconstant\hfill \\ {}\hfill 1,\wedge productconstant\hfill \\ {}\hfill 3.{3}^{\prime}\times {10}^{-2},\wedge reversableproductconstant\hfill \end{array}\right. $$

This mutational scheme allows for scaling across orders of magnitude in kinetic parameters and generates a distribution of mutational effects with a bias towards slightly degrading change that is dependent upon the activity and expression level of the protein. The mutational scheme is consistent with current thought in molecular evolution, where the range and distribution of mutational effects are influenced by the current state [22]. Most of the mutations are slightly deleterious or neutral, while advantageous mutations are rare, although slightly less so as the activity of the molecule decreases. Intuitively, as a sequence decreases in fitness contribution, the number of sequences with higher potential fitness contribution increases and as it increases in fitness contribution, the number of sequences with a higher potential fitness contribution decreases, as expected by Fisher’s geometric model.

Five different selection schemes were employed to examine the influence of various factors on pathway evolution. The first scheme involved selection on steady state flux alone, where the fitness of an individual is described below:

$$ {F}_1=\frac{1}{1+{e}^{-{\left( flux-650\right)}^{0.07}}} $$

Values in this logistic function control the asymptotic fitness and the gradient of the flux to fitness relationship. As enzymes reach limits of adaptation because of the ability to utilize products, so do pathways, where the end products are also subjected to the rules of binding and catalysis [23, 38, 39]. The asymptote of 650 and slope of 0.07 are arbitrary, but are chosen to reflect the ultimate utilizable flux. Changing them would be expected to alter the distribution of fitness effects (fraction of deleterious changes at equilibrium), but not the overall evolutionary dynamics of the system. A second (negative control) scheme was implemented to examine mutational opportunity and mutational pressure. In this experiment individuals were sampled at random from the population and only the mutational process acted.

Another control was used to examine the evolutionary stability of rate limiting steps, by implementing a scheme with selection on the first reaction rate to become rate limiting by preventing the buildup of the intermediate after the reaction, and using a neutral mutational distribution (with respect to molecular phenotype) that eliminated mutational pressure. We used the multiplicative fitness function,

$$ {F}_m={F}_1{F}_2 $$

where

$$ {F}_2=\frac{1}{e^{s\cdot \left[B\right]}} $$

Here, [B] is the concentration of the deleterious metabolite and s (9.4 × 10^−4^) is a scalar chosen to control the flux and the intersection point of the two curves. As indicated, the mean of the mutational distribution is set at 0, and the distribution is parameter-independent.

A fourth experiment was implemented to examine the role of preventing the buildup of the deleterious intermediate on pathway evolution, resulting in the same multiplicative fitness function above. This experiment used the biological (parameter dependent) mutational distribution as previously outlined. Finally, the cost of protein production was also considered using another multiplicative fitness function where *s* is a normalizing constant (1.0 x 10^−6^), cost_AA_ (30.3) and cost_nuc_ (49.2) reflect the per unit costs of synthesis [24], and enzyme lengths are given in Additional file 1: Table S2,

$$ {F}_p={F}_1{F}_3, $$

where

$$ {F}_3=\frac{1}{1+s\cdot \left( cos{t}_{protein} + cos{t}_{mRNA}\right)} $$

and

$$ \begin{array}{l} cos{t}_{protein}= cos{t}_{AA}\left\{\left[ EnzymeA\right] lengt{h}_A+\left[ EnzymeB\right] lengt{h}_B+\left[ EnzymeC\right] lengt{h}_C\right.\\ {}\left.+\left[ EnzymeD\right] lengt{h}_D+\left[ EnzymeE\right] lengt{h}_E\right\}\end{array} $$

$$ \begin{array}{l} cos{t}_{mRNA}= cos{t}_{nuc}\left\{\frac{3\cdot lengt{h}_A\cdot \left[ EnzymeA\right]}{1000}\right.+\frac{3\cdot lengt{h}_B\cdot \left[ EnzymeB\right]}{1000}\\ {}\left.+\frac{3\cdot lengt{h}_C\cdot \left[ EnzymeC\right]}{1000}+\frac{3\cdot lengt{h}_D\cdot \left[ EnzymeD\right]}{1000}+\frac{3\cdot lengt{h}_E\cdot \left[ EnzymeE\right]}{1000}\right\}\end{array} $$

Each of these simulations was run for 22,000 generations and the point of mutation-selection balance was reached by generation 20,000 under each of these selective schemes (the scheme with no selection did not reach equilibrium because there is no mutation-selection balance without selection). The point of mutation-selection balance was determined by the stability of the fitness of the median individual across generational time as assessed by observation of approximately equal rates of positive and negative changes (Additional file 1: Figures S20–S21). The point of balance was confirmed for the experiment with selection on flux alone by replicate experiments approaching the same point from lower fitnesses that were reached from higher fitnesses (Additional file 1: Figure S1).

### Identification of rate limiting steps

The sensitivity of each of the reactions across the last 2000 generations was determined by reducing each reaction rate of the median individual by 10 % while fixing the rest of the reaction rates. The difference in flux between the perturbed and unperturbed systems was used a measure of sensitivity, and the most sensitive step was determined by the reaction for which this value was the largest.

### Examination of evolution and coevolution

Examination of parameter evolution and co-evolution was based upon the values in the median individual at each generation for the 2000 generations after equilibrium was reached. Since the reversible and inhibitory reaction constants have minimal impact on the system, they were removed from the analysis. Five replicates of the same experiment were analyzed together and the rate of change of each parameter was calculated for every generation. In order to control for directional change within enzyme concentrations, catalytic, and binding constants, the average amount of change is calculated for each group and removed from each parameter within the group. 10,000 replicates were bootstrapped from this dataset by random re-sampling within each replicate and complete linkage clustering was performed using absolute correlations as a measure of relatedness between the rates of change (Additional file 1: Figures S22–S26) [40]. The largest clusters significant at the 0.05 level are used to identify co-evolving parameters.

### Simulations with variable population sizes

In order to evaluate the effects of population size on the evolutionary stability of rate-limiting steps, experiments with two different population sizes (10^2^ and 10^6^) were performed, where the small population size was a control for the results of the previous set of experiments. For this purpose, several simplifications to the procedure were made for computational tractability. In each generation, a single mutation was proposed per generation, with mutational effects and fitness as above for the experiment with selection on pathway flux. The Kimura fixation probability was used to evaluate the fixation of proposed mutations, eliminating an explicit population and any probability of multiple segregating changes. We have

$$ \psi =\frac{1-{e}^{-2c{N}_esp}}{1-{e}^{-2c{N}_es}} $$

representing the fixation probability, where N_e_ is the population size, c is the ploidy (haploid, c = 1), s is the selective coefficient (f’/f_0_-1, where f’ is the fitness after mutation and f_0_ before) and p is the initial frequency of the allele in a population. The initial frequency p was set to ½ rather than 1/N for computational efficiency, giving the property that a neutral mutation has a 50 % chance of fixation, which scales the selective coefficient. The effects of population size played out in rising from a 0.5 frequency to fixation and the introduction of new mutations was independent of population size.

The population scheme was run for 200,000 generations per experimental replicate and the rate-limiting step length was calculated as was previously described. Both population sizes were run for 30 replicates.

### Simulations with more thermodynamic realism

To evaluate the effects of biophysical constraints on the reaction landscape, simulations where mutations were constrained by Haldane’s relationship were performed for the 10^6^ population size. Although more degrees of freedom are possible with regulation, multiple substrates and products, and the involvement of cofactors, the simplest expression shows that four parameters which are non-independent as,

$$ Keq = \frac{k_{cat}*{K}_{Mr}}{k_{cat r}*{K}_M} $$

Here, K_eq_ is the equilibrium constant driven by the thermodynamics of the reaction. For this experiment, kinetic parameter initial values were set according to Haldane’s relationship (Additional file 1: Table S3)_._ To maintain the ratio, the mutational scheme was modified from that for other experiments described above. Mutations for K_Mr_ and K_M_ are drawn from a normal distribution with a mean at −1 %. The mutational effect for k_cat_is also drawn from a normal distribution with a mean at −1 % and has a modifier that is dependent on the original ratio of k_cat_ and k_catr_ and the ratio of mutated K_M_ and K_Mr_. K_catr_ is calculated from Haldane’s relationship with the mutated k_cat_, K_M_, K_Mr._ This experiment was replicated for 30 times. Rate-limiting step lengths were evaluated as was previously described.

### Characterizing allele segregation with explicit populations

In order to estimate the observed allele segregation within each population, the number of alleles for each parameter was calculated. Parameter numbers were calculated within each population for 2000 generations every 10 generations (total of 200 data points for each parameter) as the mean of total number of alleles per generation (for all parameters and for forward reaction-only parameters). Mean and standard deviation per 2000 generations were retrieved for each parameter as well as the minimum and maximum of the dataset. These values were compared with the expected allele segregation number as calculated for the population with a selectively neutral regime as previously described by Kimura and Crow [25],

$$ n=2{N}_e\mu +1 $$

Here n is the number of alleles for particular parameter, μ is mutation rate, and Ne the effective population size.

### Statistical tests and bootstrap confidence intervals

For the simulation experiments exploring the evolutionary stability of the rate limiting step, we performed permutation tests under the null hypothesis of no stability. Stability was measured by the number of consecutive generations that a reaction remained rate limiting, once it became the rate limiting step, and under the null hypothesis, each reaction should have the same average number of consecutive generations. For each of the replicates, the correspondence between each reaction and its average time spent as rate limiting was permuted, and the average absolute deviation of each reaction from the overall mean was calculated. In this manner, a null distribution was generated through 100,000 permutation replicates, and an empirical p-value was found by comparing the average absolute deviation in the actual data as compared to this null distribution.

Confidence intervals for each average number of consecutive generations that a reaction was rate limiting were constructed by first bootstrapping the replicates, and then bootstrapping the consecutive runs within each selected replicate. In this manner, a Monte Carlo sampling distribution for each average was generated, and 95 % confidence intervals were generated by taking the 2.5th and 97.5th percentiles from each bootstrap sampling distribution. Error bars in the corresponding figures reflect these confidence intervals.

To test the question of whether selection has an effect on expression cost, we examine enzyme concentration, k_cat_ and K_M_ against the length of the enzyme. Using the 2000 generations at equilibrium across the five replications and comparing the selection against flux experiment to the selection against flux and protein expression, we ran a linear mixed-effects model with random effects for the replicates, and an interaction term for enzyme length and experiment. The null hypothesis is that the interaction term is equal to 0, or in other words, that the different selection regimes have the same effect on expression cost. Due to computational burden within the mixed-effects model when attempting to account for the correlation structure induced by the Markovian nature of the simulations, only the unique values of each corresponding outcome variable were selected to be fit in the model, and standard linear mixed-effects models were run with a random effect for each replicate. While this loss of information is suboptimal, this should result in conservative inference. Assumptions of homoskedasticity and linearity of the relationship between enzyme length and parameter observations appear to be satisfied. The small effective sample size may be a concern, but should also lead to conservative inference. Statistical significance of the interaction term was assessed via likelihood ratio tests comparing the interaction model to the null model, which did not contain the interaction term.

## Reviewers’ comments

We thank the reviewers for their reviews of our manuscript. Reviewer 2 included minor comments that have not been included for publication, but have improved the readability of the manuscript.

### Reviewers’ report 1: Arne Elofsson, Stockholm University, Sweden

#### Reviewer summary

The authors describe a simulations of enzymatic reactions and identifies rate limiting steps.

#### Reviewer recommendations to author

One problem with this paper is that I am not convinced that the main reason to do this study is correct. The authors claim: “While commonly assumed in the biochemistry community that the control of metabolic pathways is thought to be critical to cellular function.”. However, they do not provide a single reference to that this really is commonly assumed, or if this is really true. Certainly it varies from pathway to pathway. It is certainly very important to have a very close control over glucose levels in the blood (see the problem for people with diabetes), while lactase efficiency can vary by many orders of magnitude without a large impact on fitness. I would interpret the results in a different way than the authors do. I do not agree with the claim “The evolutionary stability of rate limiting steps was low in all simulations with non-neutral mutational processes. ” Instead I think: If you select for positive control or against deleterious intermediates the first step is almost always rate limiting step if not it can be any step.

Author Response: *The main objective of the study is the ultimate differentiation between compensatory processes and directional selection in pathways after the characterization of the expected evolutionary dynamics of pathways with negative selection on pathway function. This is currently not well understood. There is indeed an implicit assumption of pathway stasis, from biochemistry textbooks that describe “glycolysis” and other pathways, to model organism studies that seek to transfer functions from one organism to another to GWAS and QTL studies that assume that pathway regulation and sensitivity are constant.*

*The selective regime we have applied in this study is fairly simple. It is definitely the case that the nature of the evolutionary dynamics will be altered with more complex regulatory schemes. However, these do not provide selective pressures for extra activity and evolutionarily stable control. With selection against the deleterious intermediate, there is a preponderance of cases where this step re-emerges as rate limiting more frequently than others. However, it is not stable in an evolutionary sense as rate limiting, given its short half-life in remaining flux controlling when it emerges as such. Further, the first step is only the rate limiting step when that is the step leading to the deleterious intermediate; it is not generally the first step that is rate limiting when there is a deleterious intermediate. With lactase, it is unclear to us that the excess activity in the enzyme is stable across distantly related species and we would predict otherwise.*

### Reviewers’ report 2: David Ardell, University of California- Merced, USA

#### Reviewer summary

I am not an expert on Metabolic Control Analysis (MCA) or Biochemical Systems Theory. My assessment of the present work is that it is a highly original synthesis of ideas and models that appears to have been well-implemented, that the conclusions drawn are well-founded from the results obtained, and that the results challenge apparently accepted wisdom regarding the evolutionary stability of rate-limiting steps in non-branching metabolic pathways. The review of prior literature and contextualization of results in the introduction and discussion are sufficient, yet further efforts here might benefit some readers, to dispel confusions and to better connect the results to prior work. For example, in his textbook “A First Course in Systems Biology,” E.O. Voit writes “MCA was originally conceived to replace the formerly widespread notion that every pathway has one rate-limiting step, which is a slow reaction that by itself is credited with determining the magnitude of flux through the pathway… In linear pathway sections without branches, the rate-limiting step was traditionally thought to be positioned at the first reaction, where it was possibly inhibited through feed-back exerted by the end product. In MCA, this concept of a rate-limiting step was supplanted with the concept of shared control, which posits that every step in a pathway contributes, to some degree, to the control of the steady-state flux.” In light of this, perhaps the author’s results might profitably be considered as extending MCA’s notion of distributed control in metabolic pathways to distributed selection pressures on their component enzymes coevolving on a rugged landscape. A valuable contribution made by this work, in my opinion, is the example it provides of another relatively simple biological system that under the simplest evolutionary scenarios — namely stabilizing selection on its output — explores a large neutral network of solutions (in this case, of kinetic parameters). In summary, I believe the present work is an important contribution to its field.

#### Reviewer recommendations to author

There are many points where the presentation and narrative could be improved to increase impact, especially for those unfamiliar with some among the many different disciplines and subjects touched on by this work.

Author Response: *We thank the reviewer for his summary. We have tried to improve the readability of the manuscript and to better introduce disparate concepts.*

### Reviewers’ report 3: Shamil Sunyaev, Harvard Medical School, USA

#### Reviewer summary

This manuscript challenges the idea of evolutionary stability of rate limiting steps in linear pathways. Extensive computer simulations demonstrate that rate limiting steps exist for only short evolutionary times. This is an interesting result.

#### Reviewer recommendations to authors

I find the section “Allele Segregation within Population” confusing. I suggest that the authors would clarify this section. Also, how stable are the results with respect to effect sizes and directions of incoming mutations?

Author Response: *We thank the reviewer for his summary. We have added a new introduction to the allele segregation section to improve clarity. The trajectories of effect sizes and directions of incoming mutations that are sampled for each parameter are shown in the Supplementary Figures. They derive from the mutational process described in the Methods section.*

## Abbreviations

[E], [Enzyme], the concentration of the enzyme; k_cat_, catalytic constant; K_I_, inhibition constant; K_M_, Michaelis constant; mRNA, messenger ribonucleic acid; N_e_, effective population size; μ, mutation rate.

## Additional file

Additional file 1: Table S1. The initial values given to parameters in the system at the start of each evolutionary simulation where equilibrium is approached from above are shown. **Table S2.** The lengths of each enzyme, given in the number of amino acids, are shown. **Table S3.** The initial values given to kcat, kcatr, KM and KMr parameters in the system at the start of the evolutionary simulation when constrained with Haldane’s relationship are shown. Keq and ΔG0 for each reaction are also shown. **Figure S1.** The fitness value of the median individual demonstrating that the same point of mutation-selection balance is reached when simulations begin at a lower fitness. **Figures S2–S4.** The evolution of parameter values for the experiment which started from a lower fitness are shown. **Figures S5–S19.** The averaged median of parameters after the point of mutation-selection balance is shown. **Figure S20.** The rate of change in averaged median fitness across each of the simulations is shown for A) mutation only, B) selection on flux alone, C) selection on flux and against total expression cost, D) selection on flux and against a high concentration of a deleterious intermediate, and E) non-biological neutral mutation, selection on flux, and for the first reaction to be rate limiting. Blue denotes a positive rate of change and red denotes a negative rate of change. **Figure S21.** Average median fitness across each of the simulations is shown for A) mutation only, B) selection on flux alone, C) selection on flux and against total expression cost, D) selection on flux and against a high concentration of a deleterious intermediate, and E) non-biological neutral mutation, selection on flux, and for the first reaction to be rate limiting. **Figures S22–S26.** Complete linkage clustering of parameter values for each selective scheme are shown, resulting in the data in Fig. 4. (PDF 1185 kb)
