# Supplementary material for: Selection on metabolic pathway function in the presence of mutation-selection-drift balance leads to rate-limiting steps that are not evolutionarily stable
Source: Biol Direct. 2016 Jul 8;11:31. doi: 10.1186/s13062-016-0133-6 (PMC4938953; doi:10.1186/s13062-016-0133-6)
Supplement: Additional file 1: Table S1. — The initial values given to parameters in the system at the start of each evolutionary simulation where equilibrium is approached from above are shown. Table S2. The lengths of each enzyme, given in the number of amino acids, are shown. Table S3. The initial values given to kcat, kcatr, KM and KMr parameters in the system at the start of the evolutionary simulation when constrained with Haldane’s relationship are shown. Keq and ΔG0 for each reaction are also shown. Figure S1. The fitness value of the median individual demonstrating that the same point of mutation-selection balance is reached when simulations begin at a lower fitness. Figures S2–S4. The evolution of parameter values for the experiment which started from a lower fitness are shown. Figures S5–S19. The averaged median of parameters after the point of mutation-selection balance is shown. Figure S20. The rate of change in averaged median fitness across each of the simulations is shown for A) mutation only, B) selection on flux alone, C) selection on flux and against total expression cost, D) selection on flux and against a high concentration of a deleterious intermediate, and E) non-biological neutral mutation, selection on flux, and for the first reaction to be rate limiting. Blue denotes a positive rate of change and red denotes a negative rate of change. Figure S21. Average median fitness across each of the simulations is shown for A) mutation only, B) selection on flux alone, C) selection on flux and against total expression cost, D) selection on flux and against a high concentration of a deleterious intermediate, and E) non-biological neutral mutation, selection on flux, and for the first reaction to be rate limiting. Figures S22–S26. Complete linkage clustering of parameter values for each selective scheme are shown, resulting in the data in Fig. 4. (PDF 1185 kb) [file 13062_2016_133_MOESM1_ESM.pdf]

Supplemental Materials, Orlenko et al.

Supplemental Table Legends

Table 1. The initial values given to parameters in the system at the start of each evolutionary simulation where equilibrium is approached from above are shown.

Table 2. The lengths of each enzyme, given in the number of amino acids, are shown. This is used in the calculation of the expression cost for the experiment where that is relevant.

Table 3. The initial values given to k_{cat}, k_{catr}, K_M and K_{Mr} parameters in the system at the start of the evolutionary simulation when constrained with Haldane's relationship are shown. K_{eq} and ΔG^0 for each reaction are also shown.

Supplemental Figure Legends

Figure 1. The fitness value of the median individual demonstrating that the same point of mutation-selection balance is reached when simulations begin at a lower fitness. Dashed lines represent the minimum and maximum fitnesses across replicates achieved by selection on flux when simulations began at a higher fitness.

Figure 2-4. The evolution of parameter values for the experiment which started from a lower fitness are shown. Parameters were initialized with values 5% below (5% above in the case of K_M) the averaged across-replicates parameter value of the median individual over 2000 generations post equilibrium (generations 20000-22000) from the experiment in which flux alone was considered in the selective scheme. Error bars delineate the maximum and minimum values found for each of the parameters when starting from a higher fitness.

Figure 5-19. The averaged median of parameters after the point of mutation-selection balance is shown.

Figure 20. The rate of change in averaged median fitness across each of the simulations is shown for A) mutation only, B) selection on flux alone, C) selection on flux and against total expression cost, D) selection on flux and against a high concentration of a deleterious intermediate, and E) non-biological neutral mutation, selection on flux, and for the first reaction to be rate limiting. Blue denotes a positive rate of change and red denotes a negative rate of change.

Figure 21. Average median fitness across each of the simulations is shown for A) mutation only, B) selection on flux alone, C) selection on flux and against total expression cost, D) selection on flux and against a high concentration of a deleterious intermediate, and E) non-biological neutral mutation, selection on flux, and for the first reaction to be rate limiting. The black line is the average fitness across time. Blue denotes an increase from the average fitness and red denotes a decrease from the average fitness.

Figure 22-26. Complete linkage clustering of parameter values for each selective scheme are shown, resulting in the data in Figure 4. Approximate unbiased (au) p-values computed from multiscale bootstrap resampling and bootstrap probabilities (bp) are given (see Methods and [20]). Clusters with au larger than 95% are shown in rectangles.

Table 1

**Parameter**	**Initial Value**
[Enzyme]	10 mmol/l
k_{cat}	100 mmol/l/s
k_{catr}	3000 mmol/l/s
K_M	0.1 mmol/l
K_{Mr}	30 mmol/l
K_i	40 mmol/l

Table 2

**Enzyme**	**Number of amino acids**
A	433
B	987
C	416
D	73
E	506

Table 3.

	k_{cat}, mmol/l/s	k_{catr}, mmol/l/s	K_M, mmol/l	K_{Mr}, mmol/l/s	K_{eq}	ΔG^0, kJ/mol
Reaction 1	300	3000	0.01	30	300	-14.14
Reaction 2	100	3000	0.1	30	10	-5.71
Reaction 3	75	3000	1.0	30	0.75	0.71
Reaction 4	75	3000	1.5	30	0.5	1.72
Reaction 5	200	3000	0.001	30	2000	-18.84

Figure 1

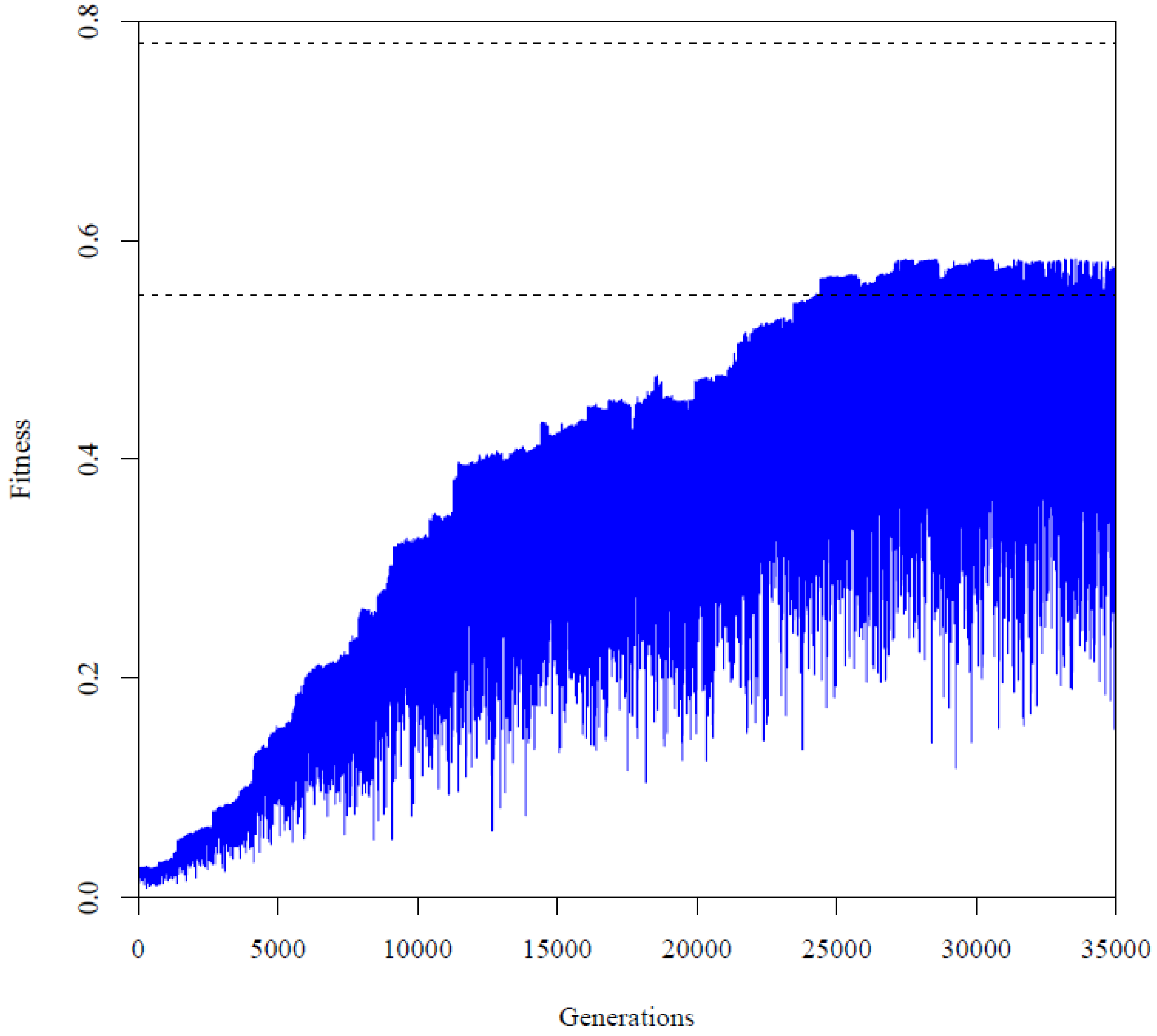

0.8
0.6
0.4
0.2
0.0
Fitness
0
5000
10000
15000
20000
25000
30000
35000
Generations

Figure 2

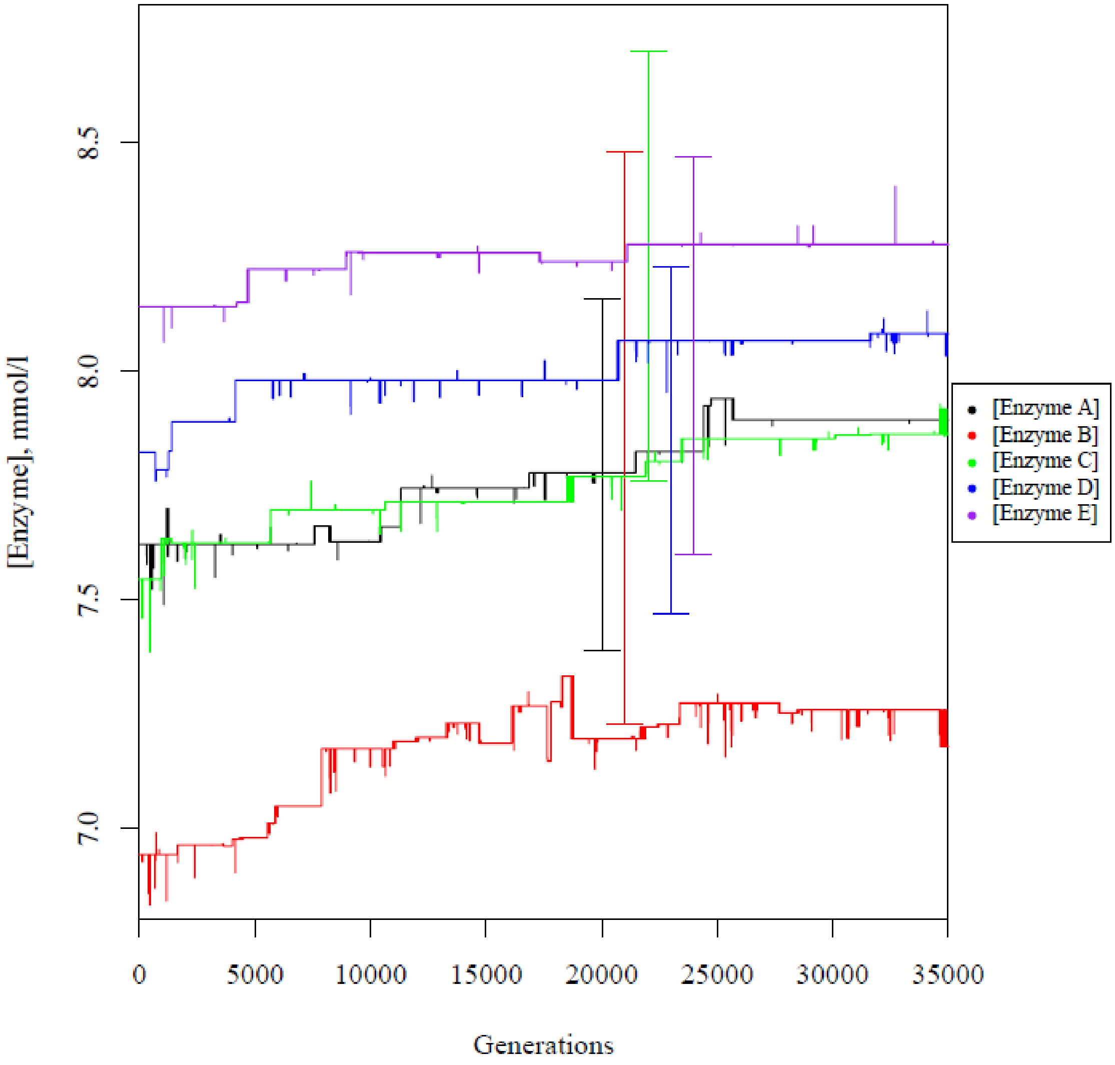

8.5
8.0
7.5
7.0
[Enzyme], mmol/l
0
5000
10000
15000
20000
25000
30000
35000
Generations
[Enzyme A]
[Enzyme B]
[Enzyme C]
[Enzyme D]
[Enzyme E]

Figure 3

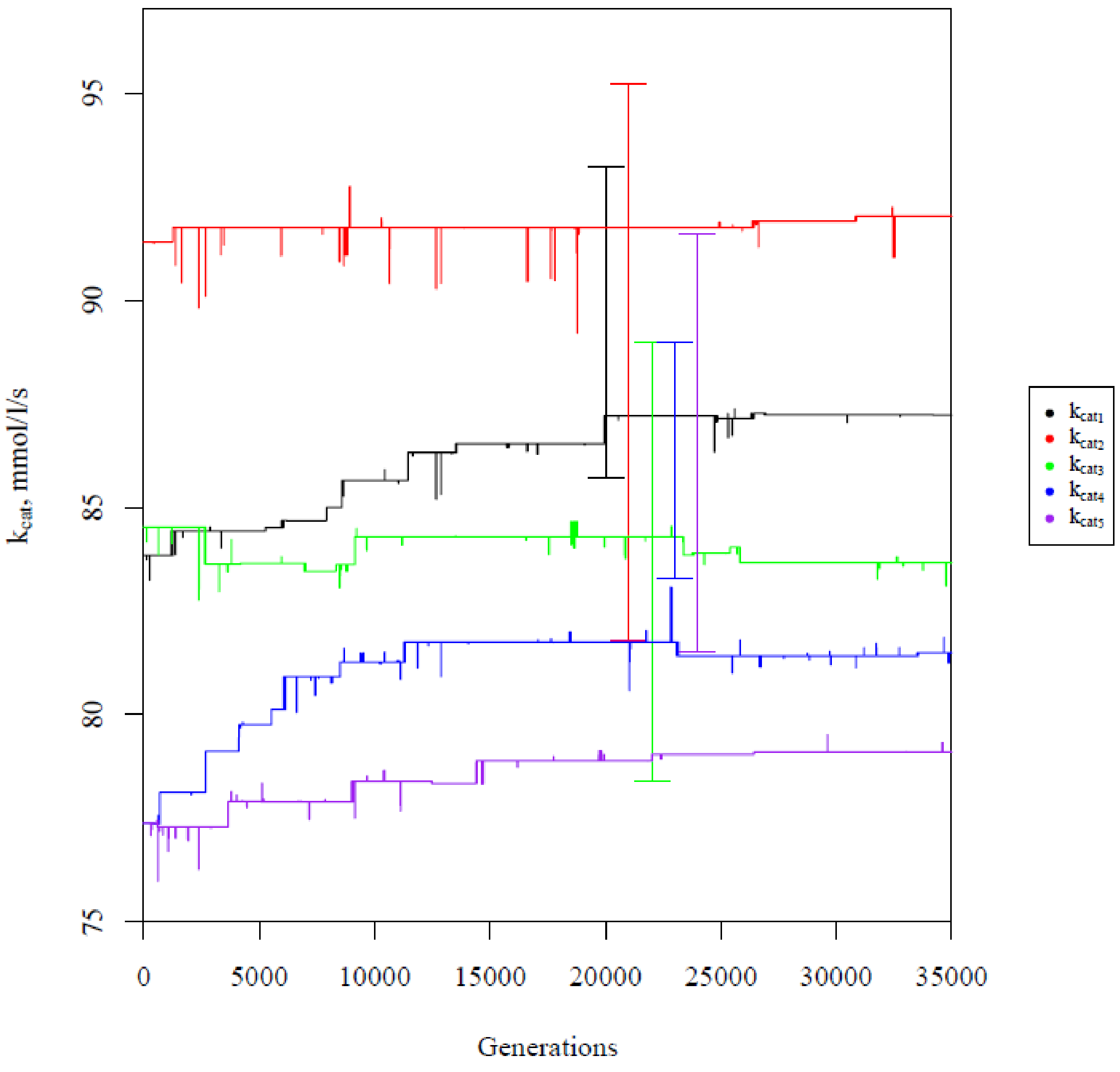

95
90
85
80
75
k_{cat}, mmol/l/s
0
5000
10000
15000
20000
25000
30000
35000
Generations
k_{cat1}
k_{cat2}
k_{cat3}
k_{cat4}
k_{cat5}

Figure 4

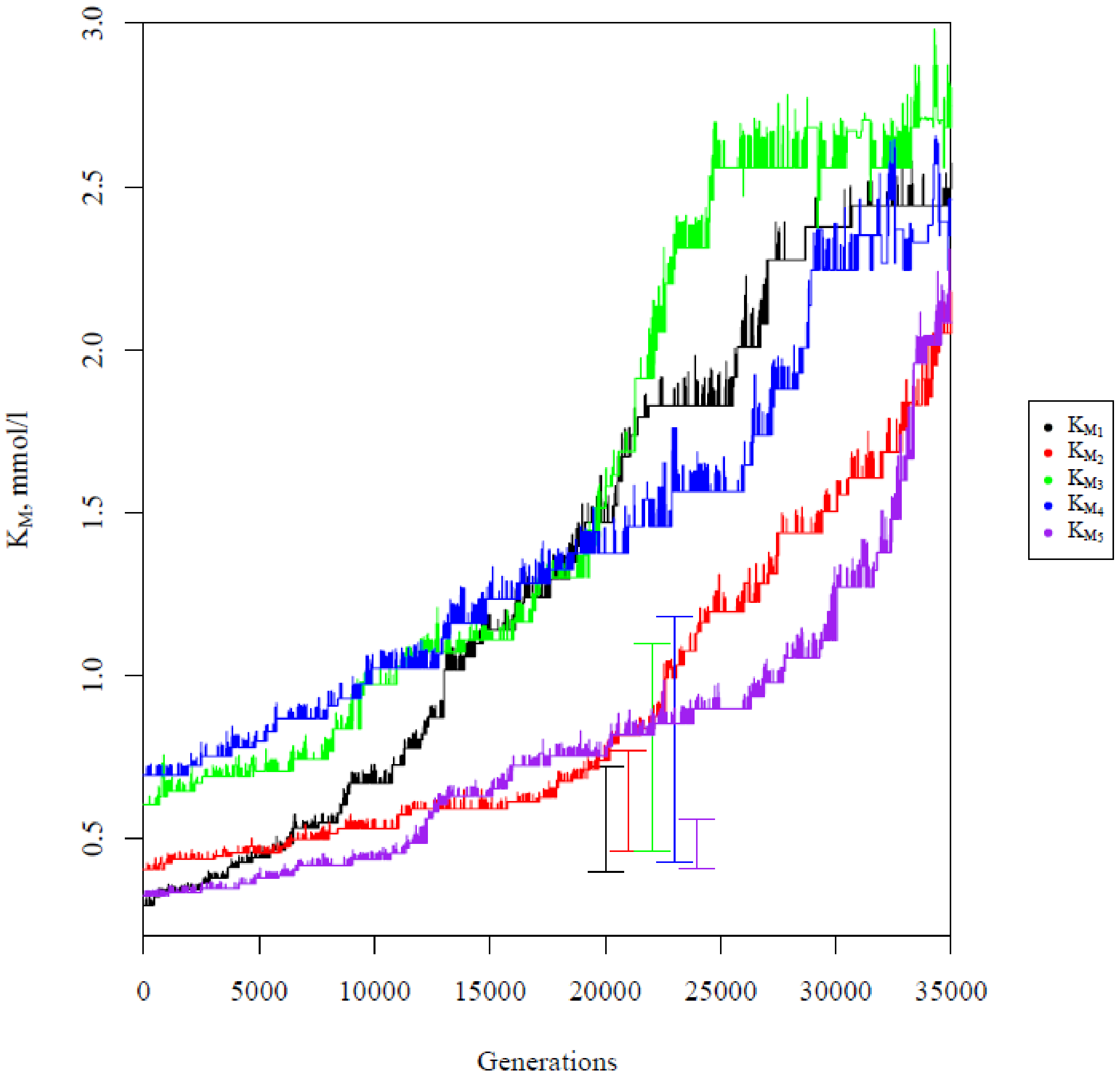

K_M, mmol/l
3.0
2.5
2.0
1.5
1.0
0.5
0
5000
10000
15000
20000
25000
30000
35000
Generations
K_{M_1}
K_{M_2}
K_{M_3}
K_{M_4}
K_{M_5}

Figure 5

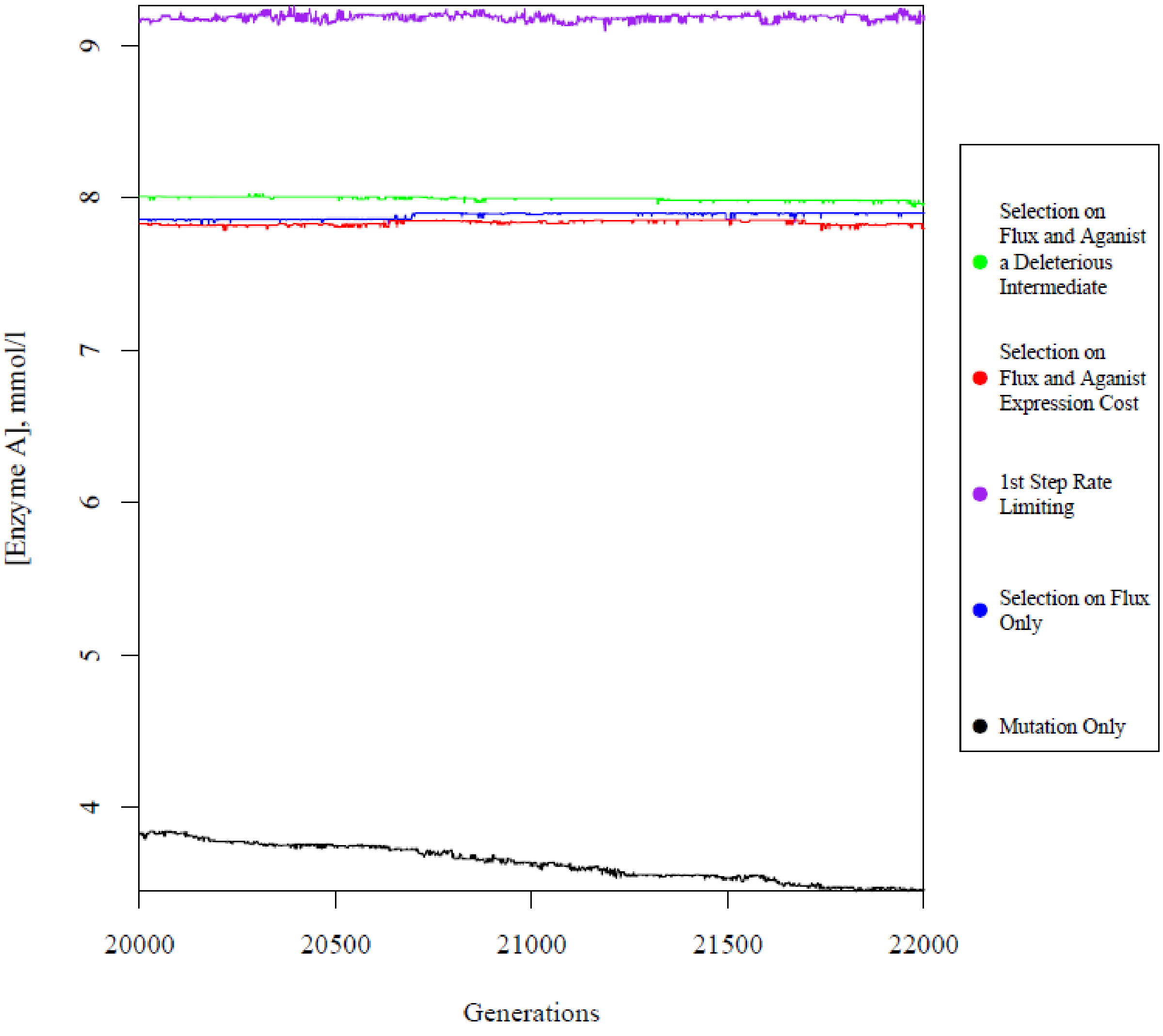

[Enzyme A], mmol/l
9
8
7
6
5
4
20000
20500
21000
21500
22000
Generations
Selection on Flux and Aganist a Deleterious Intermediate
Selection on Flux and Aganist Expression Cost
1st Step Rate Limiting
Selection on Flux Only
Mutation Only

Figure 6

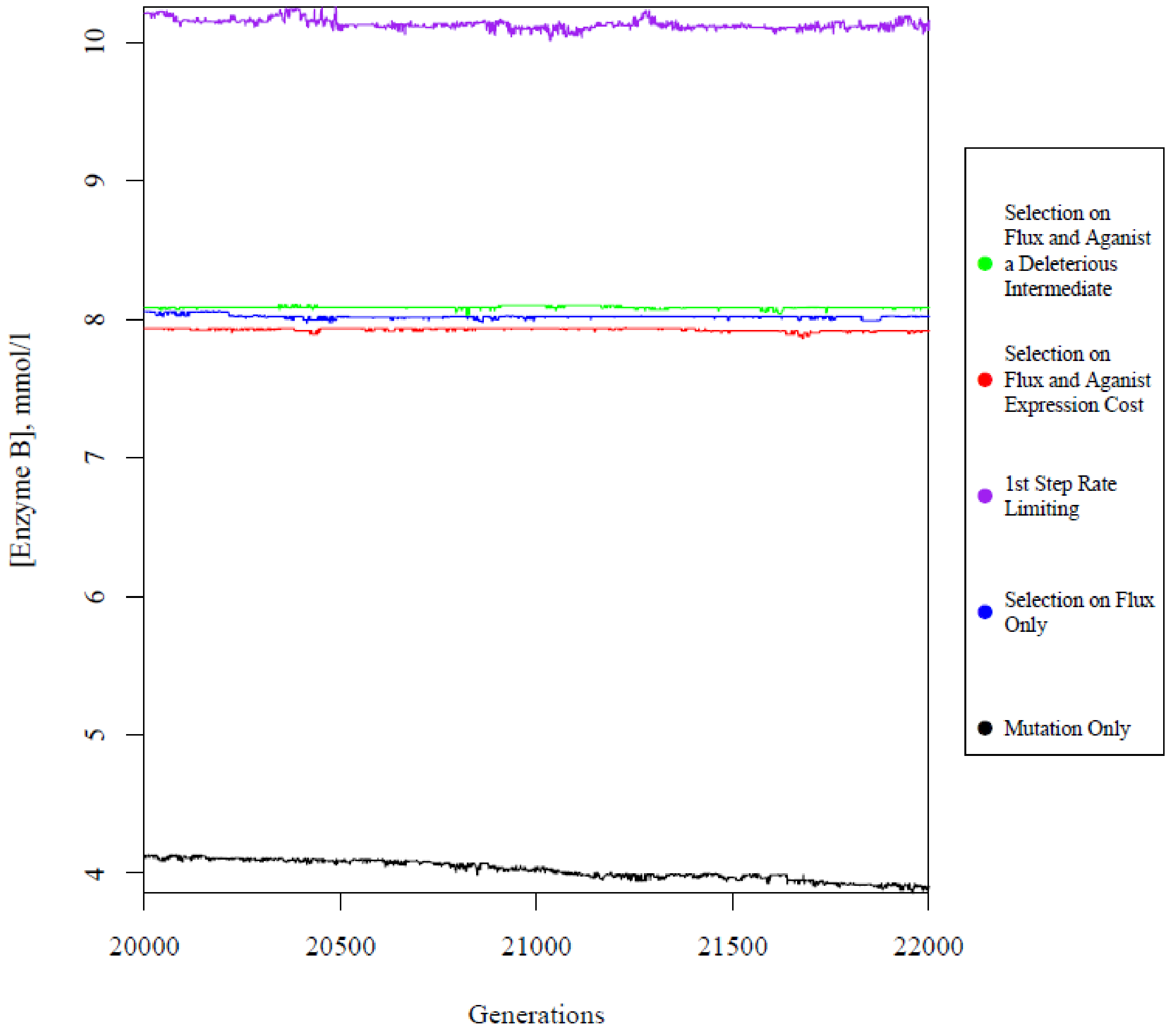
[Enzyme B], mmol/l
10
9
8
7
6
5
4
20000
20500
21000
21500
22000
Generations
Selection on Flux and Aganist a Deleterious Intermediate
Selection on Flux and Aganist Expression Cost
1st Step Rate Limiting
Selection on Flux Only
Mutation Only

Figure 7

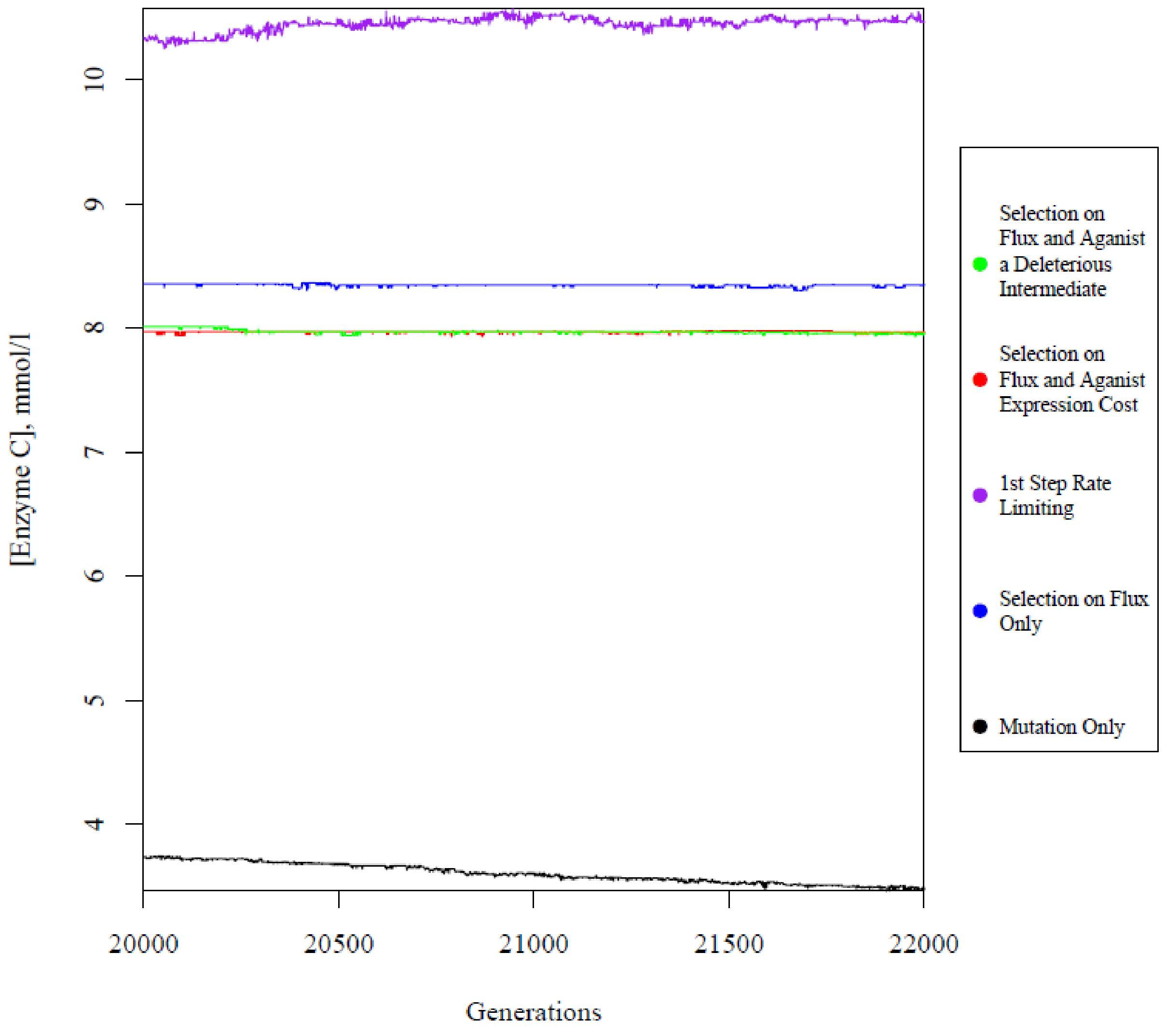

[Enzyme C], mmol/l
10
9
8
7
6
5
4
20000
20500
21000
21500
22000
Generations
Selection on Flux and Aganist a Deleterious Intermediate
Selection on Flux and Aganist Expression Cost
1st Step Rate Limiting
Selection on Flux Only
Mutation Only

Figure 8

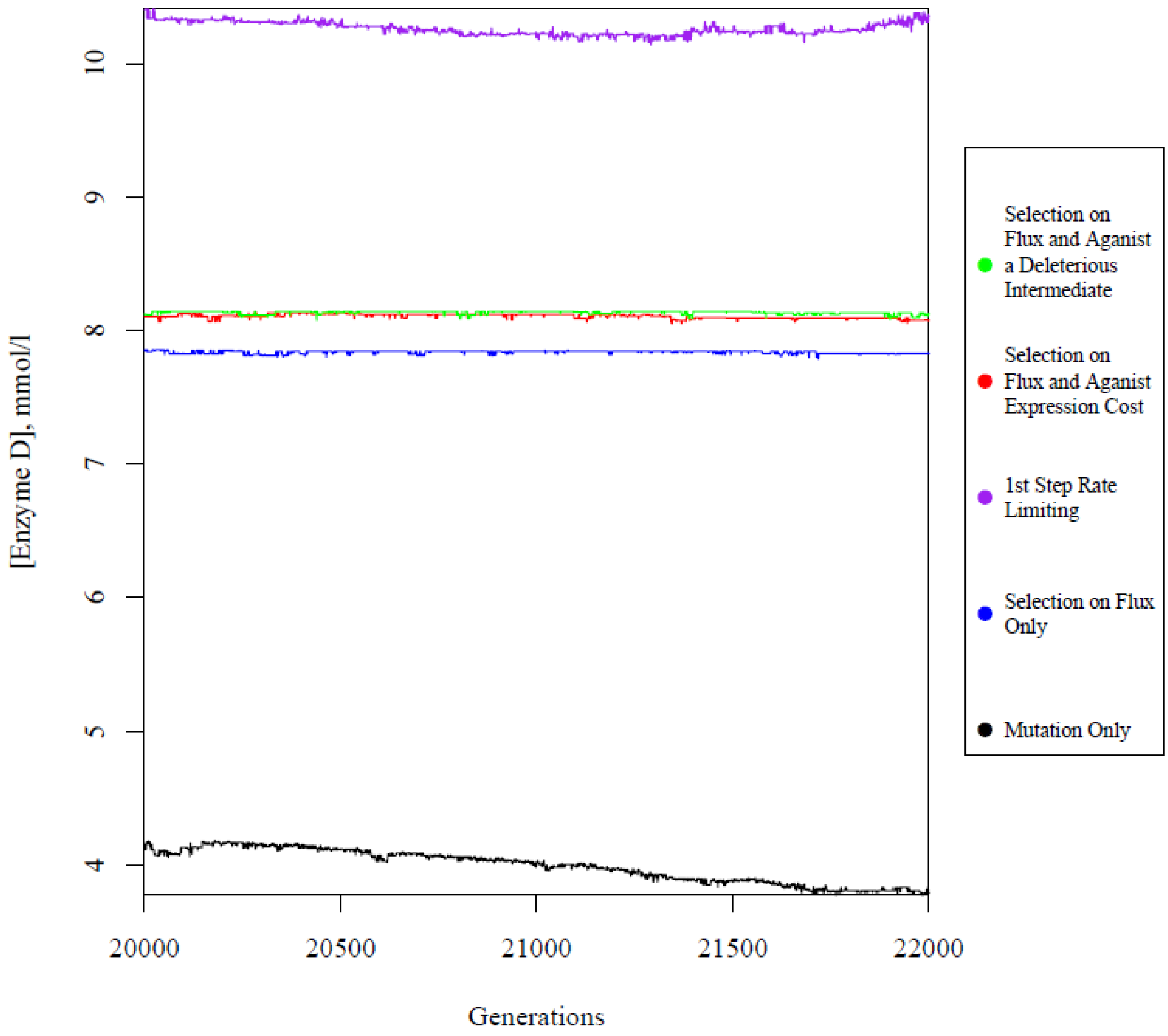
10
9
8
7
6
5
4
[Enzyme D], mmol/l
20000
20500
21000
21500
22000
Generations
Selection on Flux and Aganist a Deleterious Intermediate
Selection on Flux and Aganist Expression Cost
1st Step Rate Limiting
Selection on Flux Only
Mutation Only

Figure 9

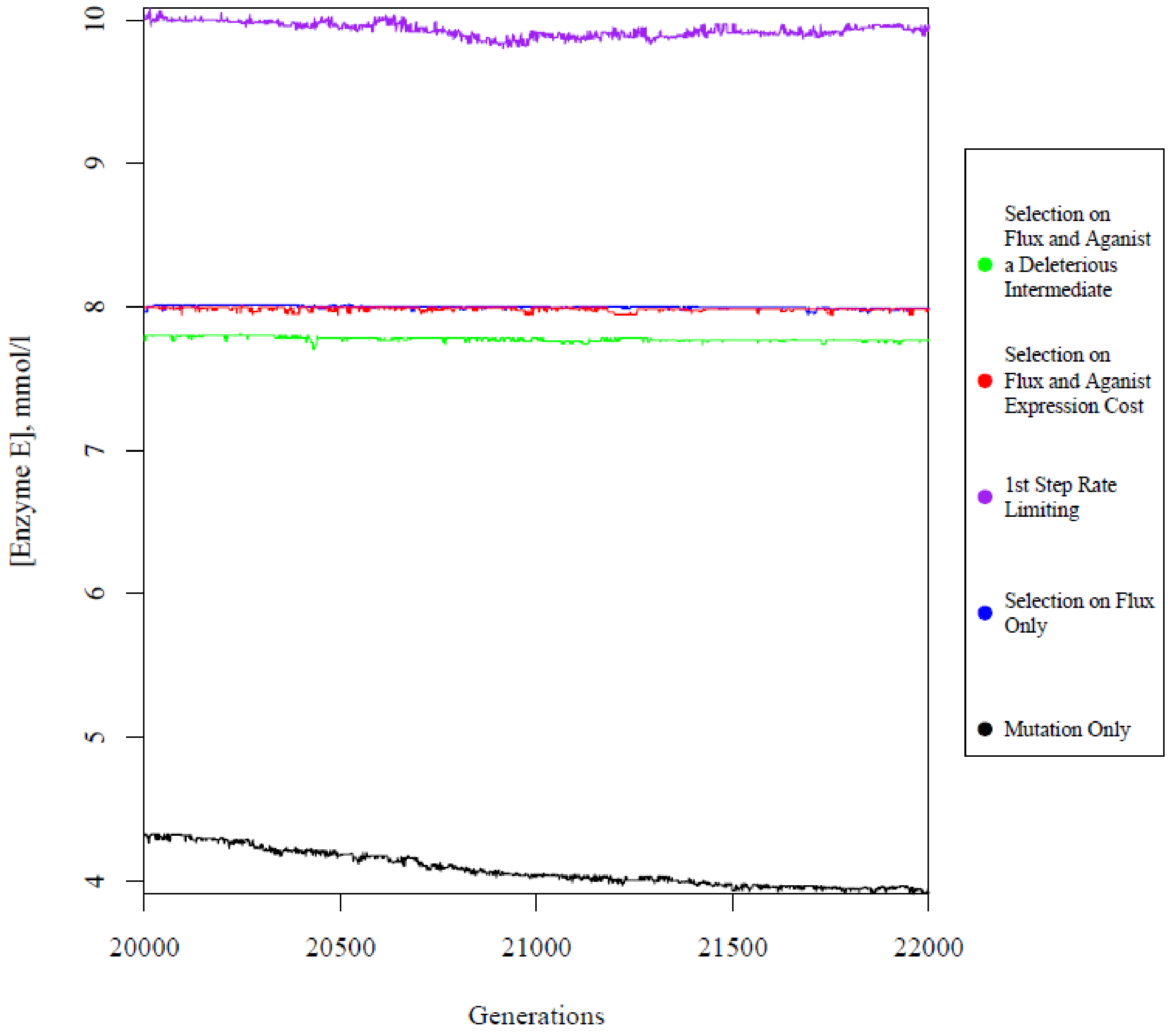
10
9
8
7
6
5
4
[Enzyme E], mmol/l
20000
20500
21000
21500
22000
Generations
Selection on Flux and Aganist a Deleterious Intermediate
Selection on Flux and Aganist Expression Cost
1st Step Rate Limiting
Selection on Flux Only
Mutation Only

Figure 10

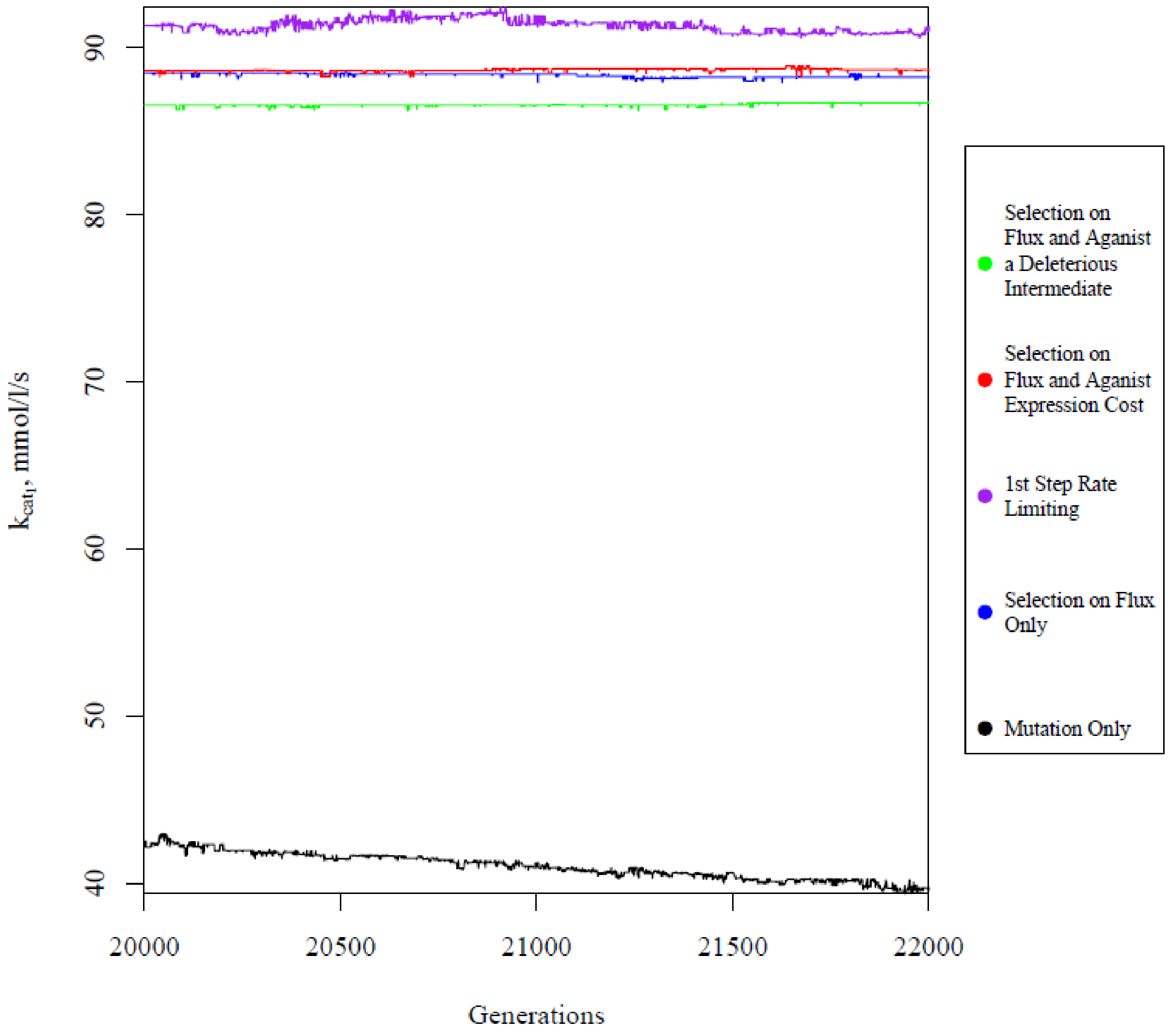

k_{cat_1}, mmol/l/s
90
80
70
60
50
40
20000
20500
21000
21500
22000
Generations
Selection on Flux and Aganist a Deleterious Intermediate
Selection on Flux and Aganist Expression Cost
1st Step Rate Limiting
Selection on Flux Only
Mutation Only

Figure 11

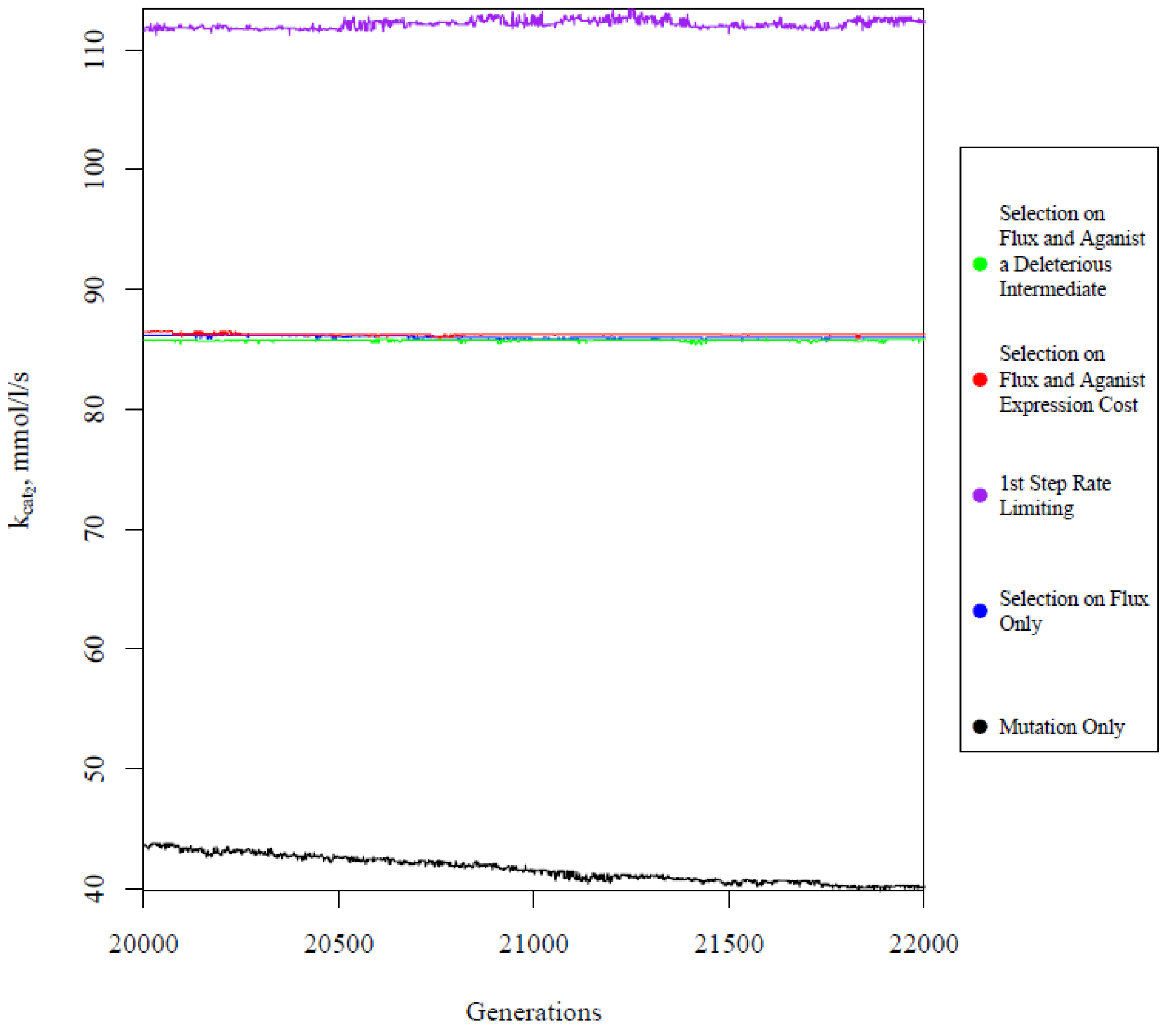

110
100
90
80
70
60
50
40
k_{cat}, mmol/l/s
20000
20500
21000
21500
22000
Generations
Selection on Flux and Aganist a Deleterious Intermediate
Selection on Flux and Aganist Expression Cost
1st Step Rate Limiting
Selection on Flux Only
Mutation Only

Figure 12

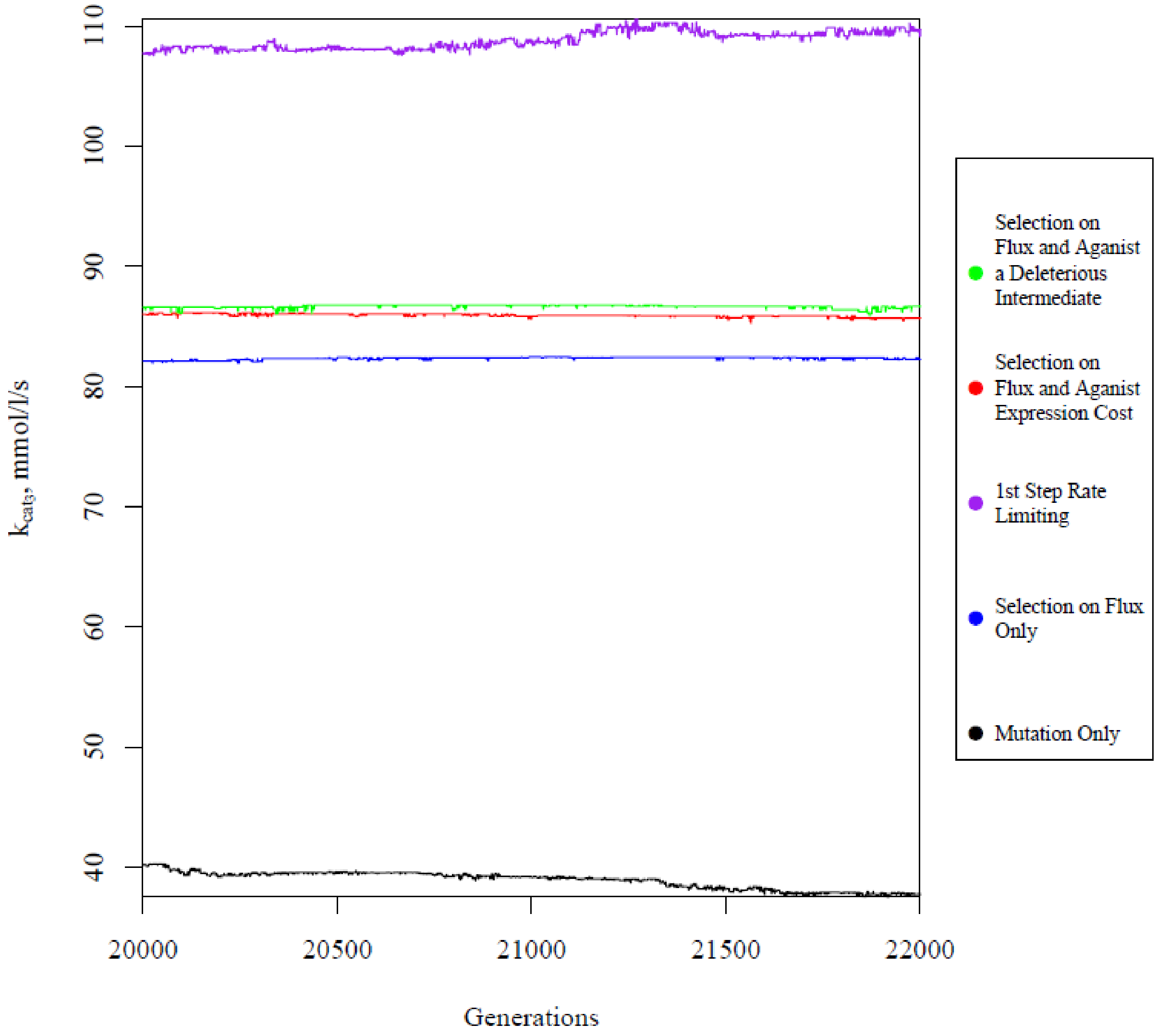

110
100
90
80
70
60
50
40
k_{cat_3}, mmol/l/s
20000
20500
21000
21500
22000
Generations
Selection on Flux and Aganist a Deleterious Intermediate
Selection on Flux and Aganist Expression Cost
1st Step Rate Limiting
Selection on Flux Only
Mutation Only

Figure 13

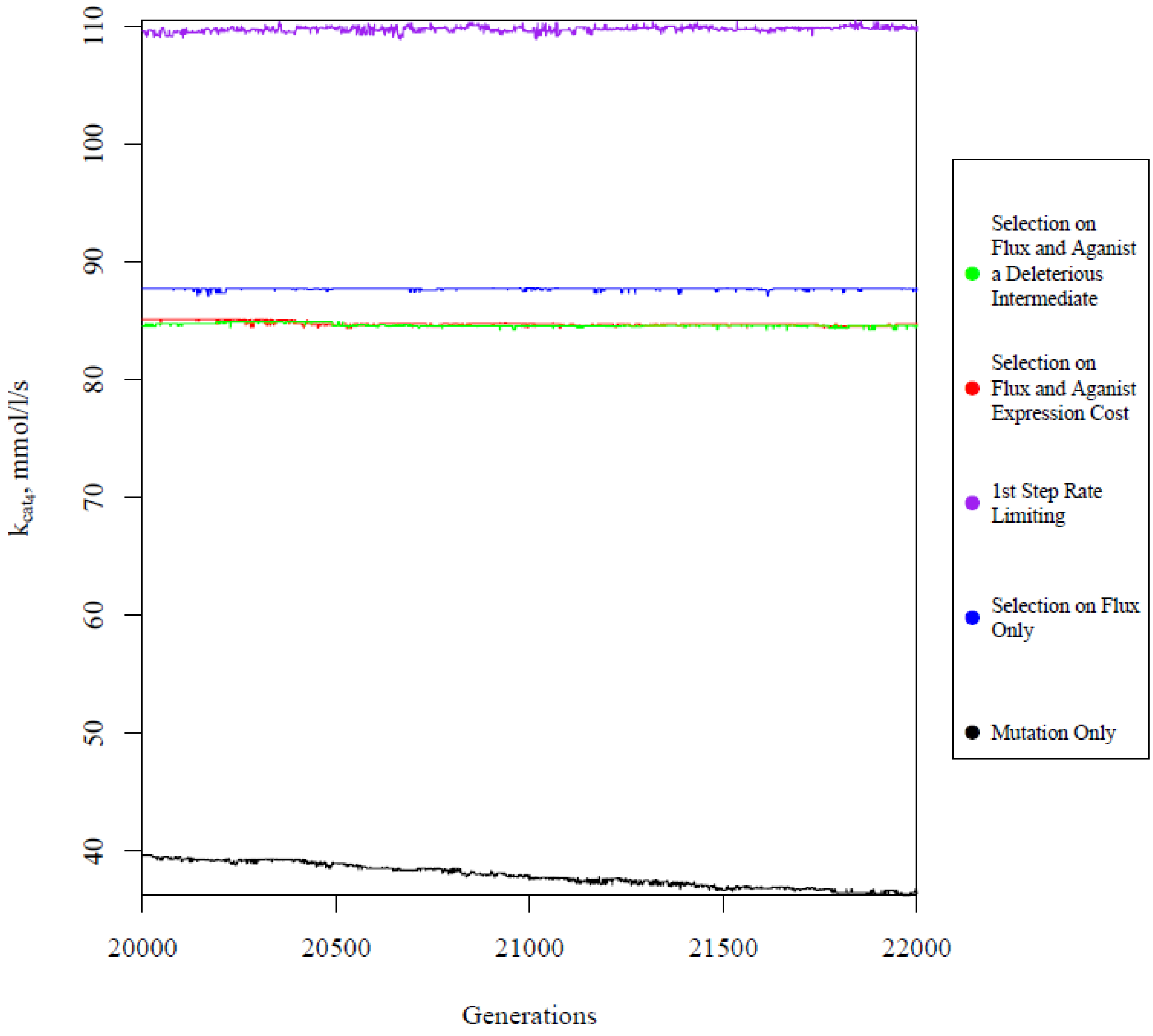

110
100
90
80
70
60
50
40
k_{cat_4}, mmol/l/s
20000
20500
21000
21500
22000
Generations
Selection on Flux and Aganist a Deleterious Intermediate
Selection on Flux and Aganist Expression Cost
1st Step Rate Limiting
Selection on Flux Only
Mutation Only

Figure 14

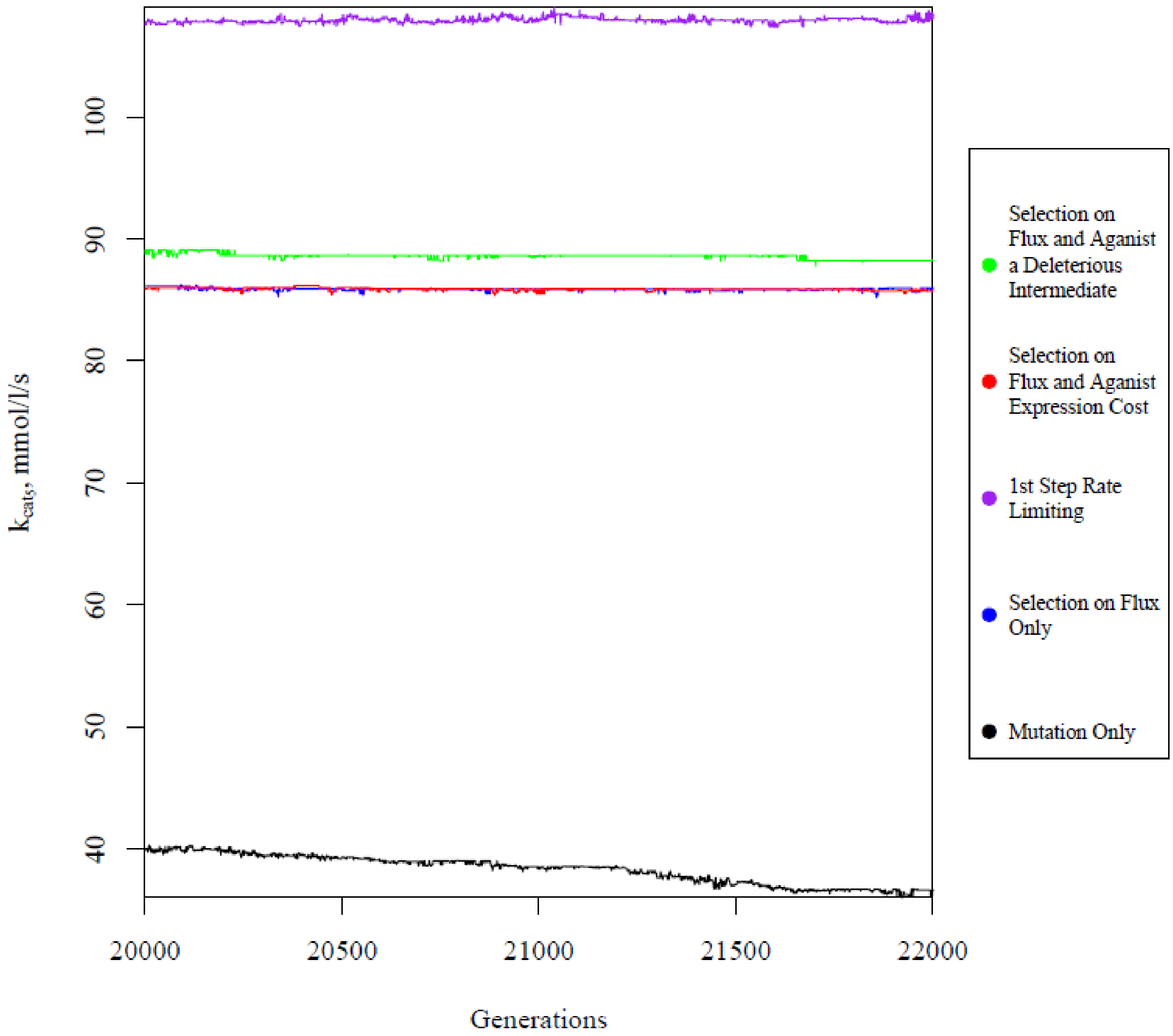

k_{cat_3}, mmol/l/s
100
90
80
70
60
50
40
20000
20500
21000
21500
22000
Generations
Selection on Flux and Aganist a Deleterious Intermediate
Selection on Flux and Aganist Expression Cost
1st Step Rate Limiting
Selection on Flux Only
Mutation Only

Figure 15

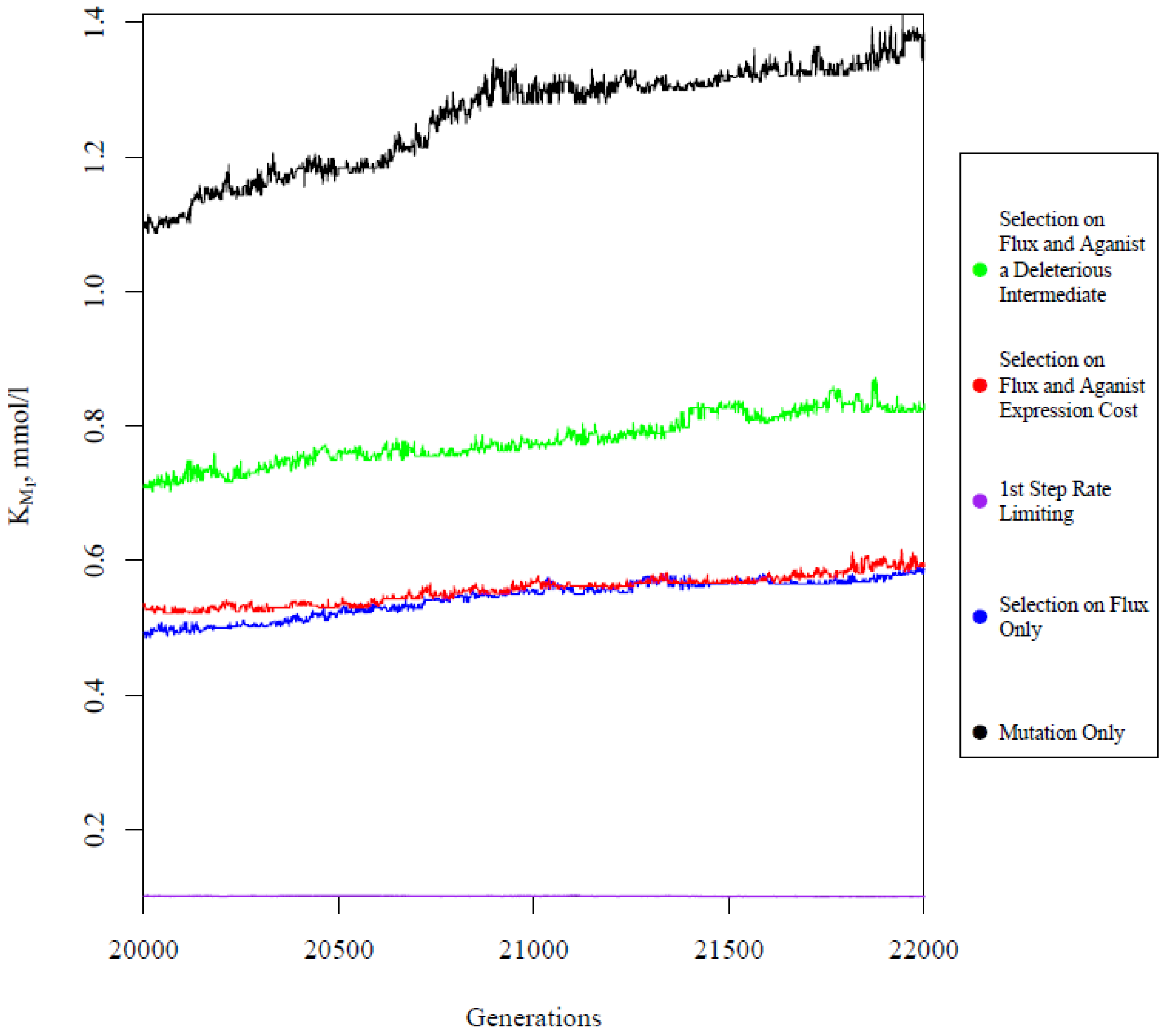

1.4
1.2
1.0
0.8
0.6
0.4
0.2
K_{MI}, mmol/l
20000
20500
21000
21500
22000
Generations
Selection on Flux and Aganist a Deleterious Intermediate
Selection on Flux and Aganist Expression Cost
1st Step Rate Limiting
Selection on Flux Only
Mutation Only

Figure 16

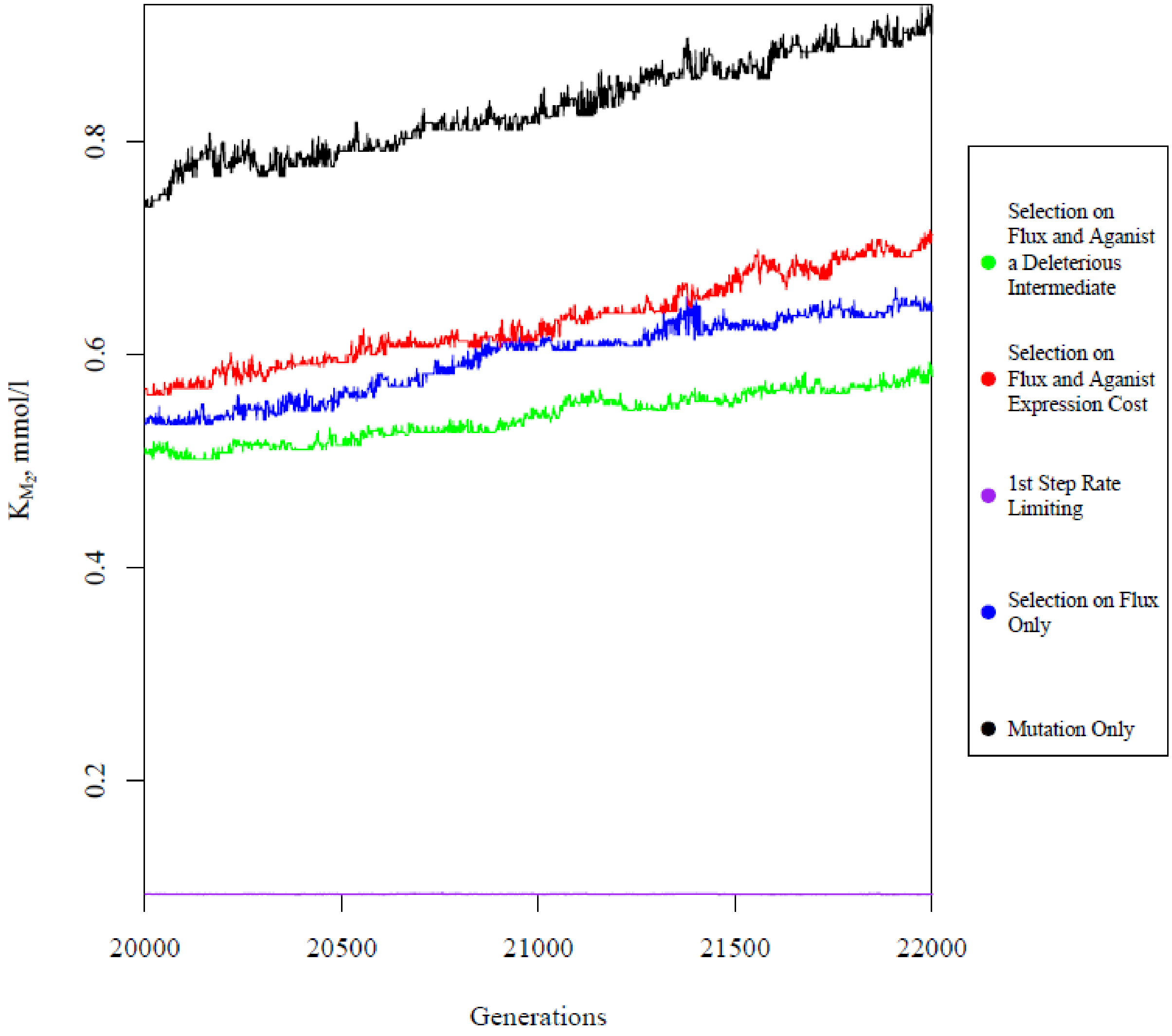

0.2
0.4
0.6
0.8
K_{M_2}, mmol/l
20000
20500
21000
21500
22000
Generations
Selection on Flux and Aganist a Deleterious Intermediate
Selection on Flux and Aganist Expression Cost
1st Step Rate Limiting
Selection on Flux Only
Mutation Only

Figure 17

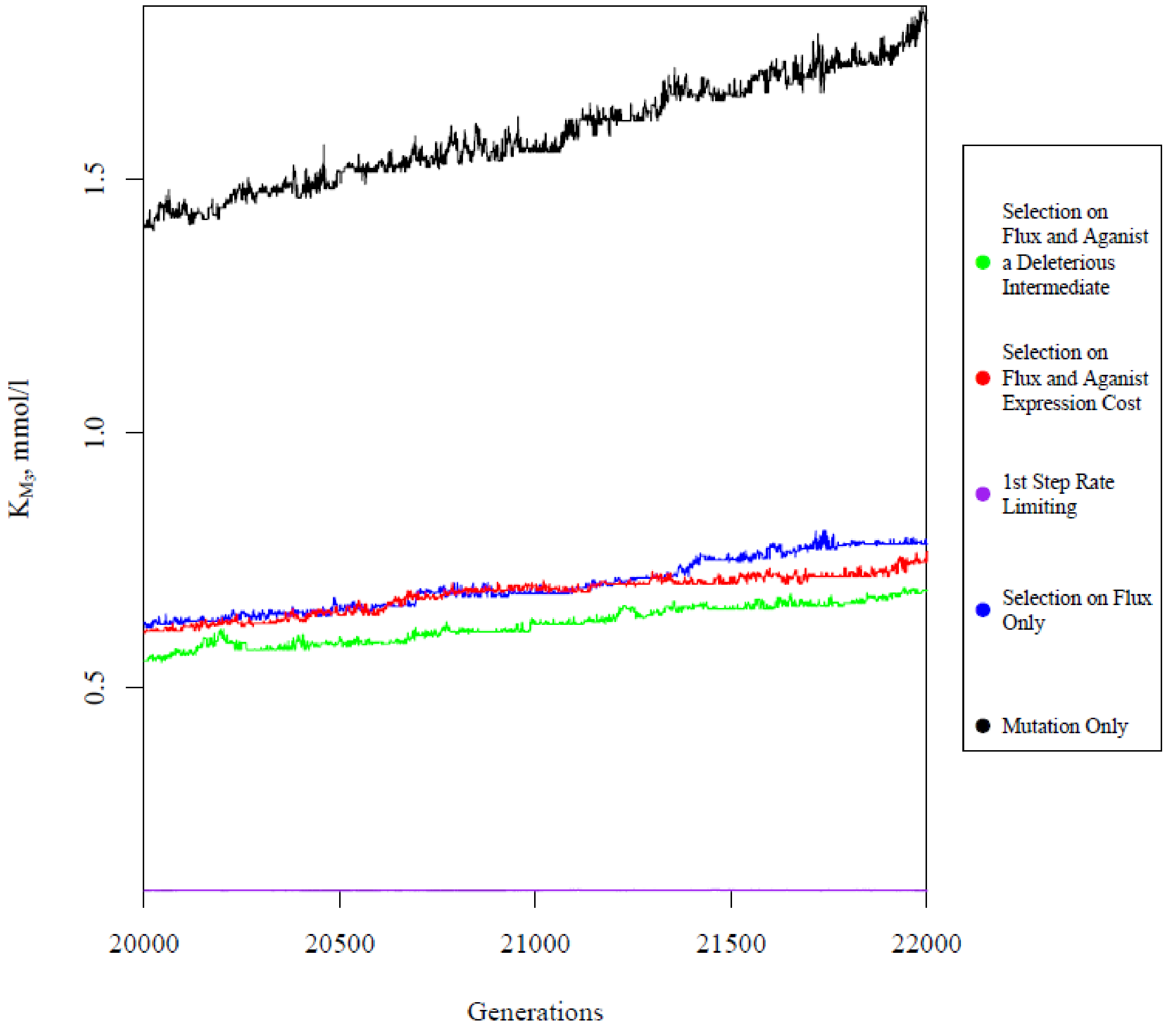

1.5
1.0
0.5
K_{M_3}, mmol/l
20000
20500
21000
21500
22000
Generations
Selection on Flux and Aganist a Deleterious Intermediate
Selection on Flux and Aganist Expression Cost
1st Step Rate Limiting
Selection on Flux Only
Mutation Only

Figure 18

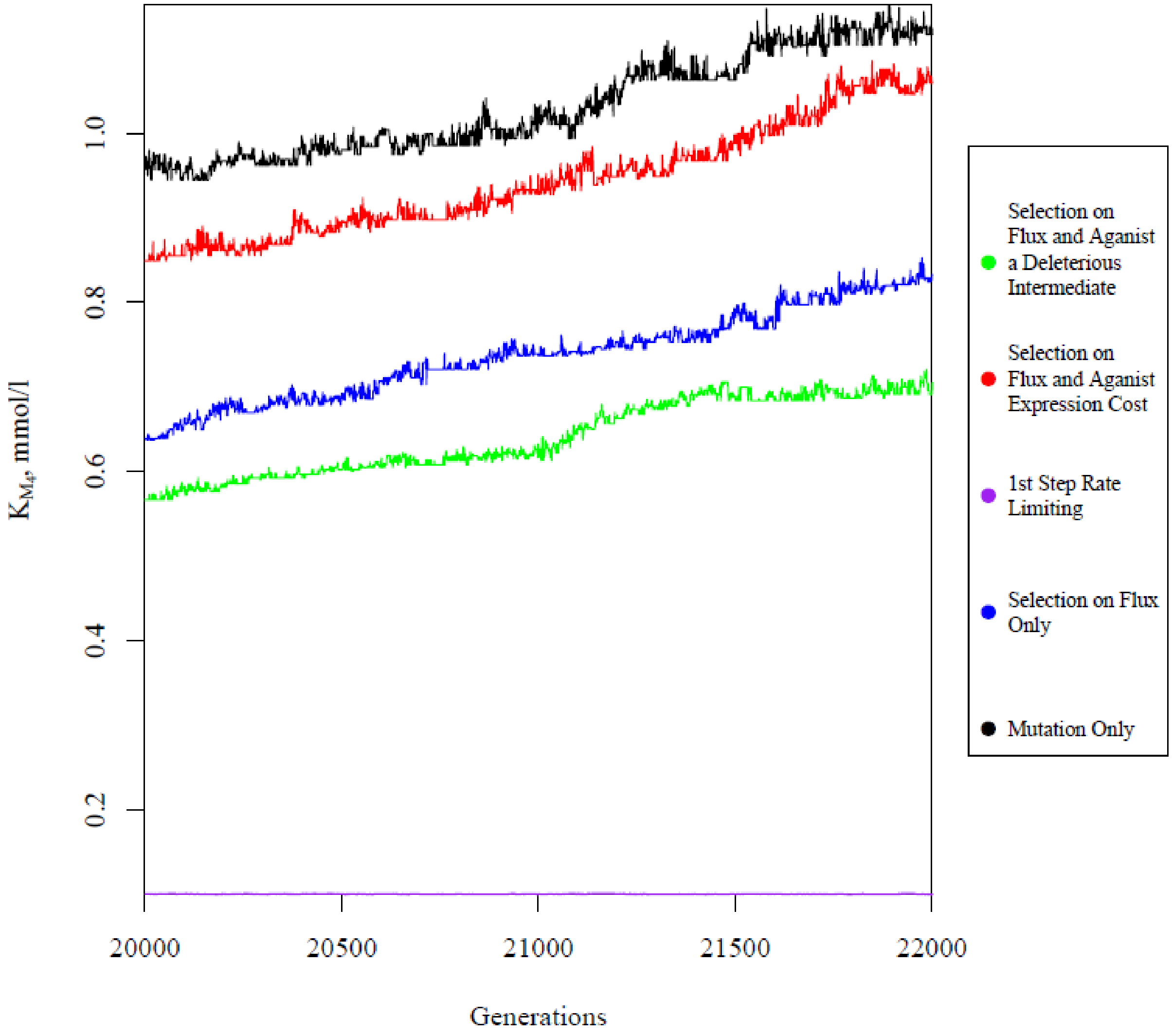

K_{M_4}, mmol/l
1.0
0.8
0.6
0.4
0.2
20000
20500
21000
21500
22000
Generations
Selection on Flux and Aganist a Deleterious Intermediate
Selection on Flux and Aganist Expression Cost
1st Step Rate Limiting
Selection on Flux Only
Mutation Only

Figure 19

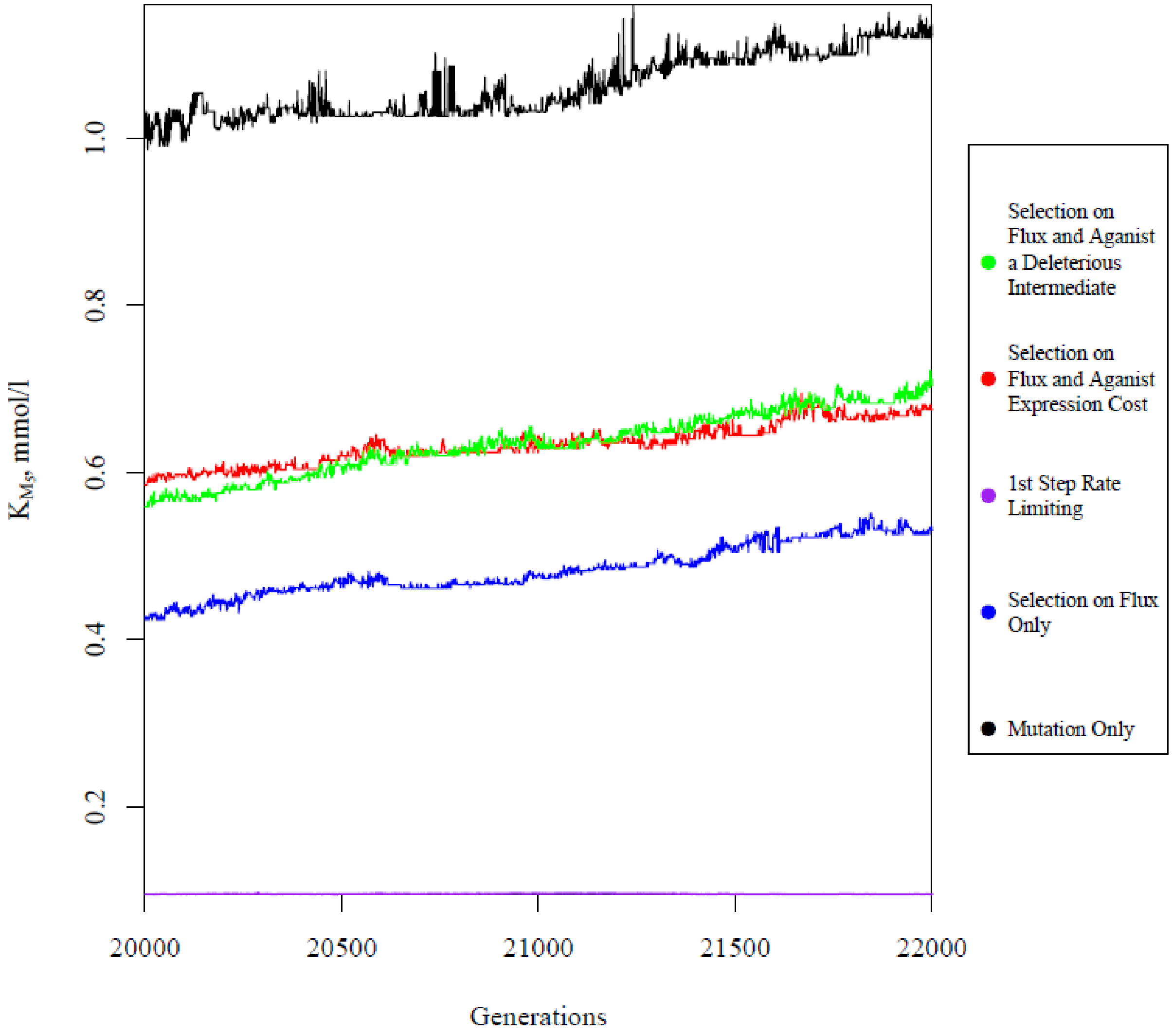

1.0
0.8
0.6
0.4
0.2
K_{Ms}, mmol/l
20000
20500
21000
21500
22000
Generations
Selection on Flux and Aganist a Deleterious Intermediate
Selection on Flux and Aganist Expression Cost
1st Step Rate Limiting
Selection on Flux Only
Mutation Only

Figure 20

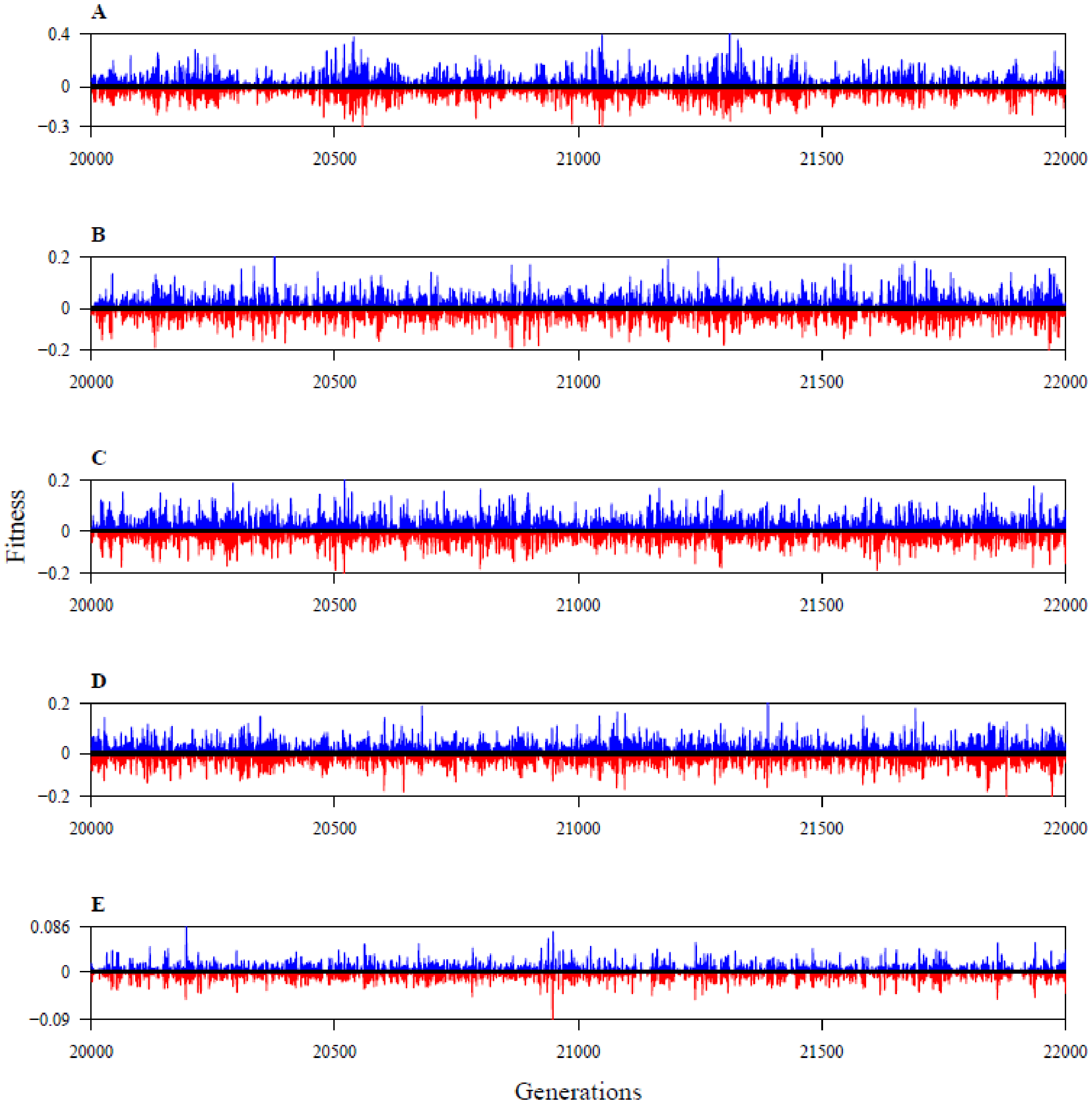
A
0.4
0
−0.3
20000
20500
21000
21500
22000
B
0.2
0
−0.2
C
0.2
0
−0.2
D
0.2
0
−0.2
E
0.086
0
−0.09
Fitness
Generations

Figure 21

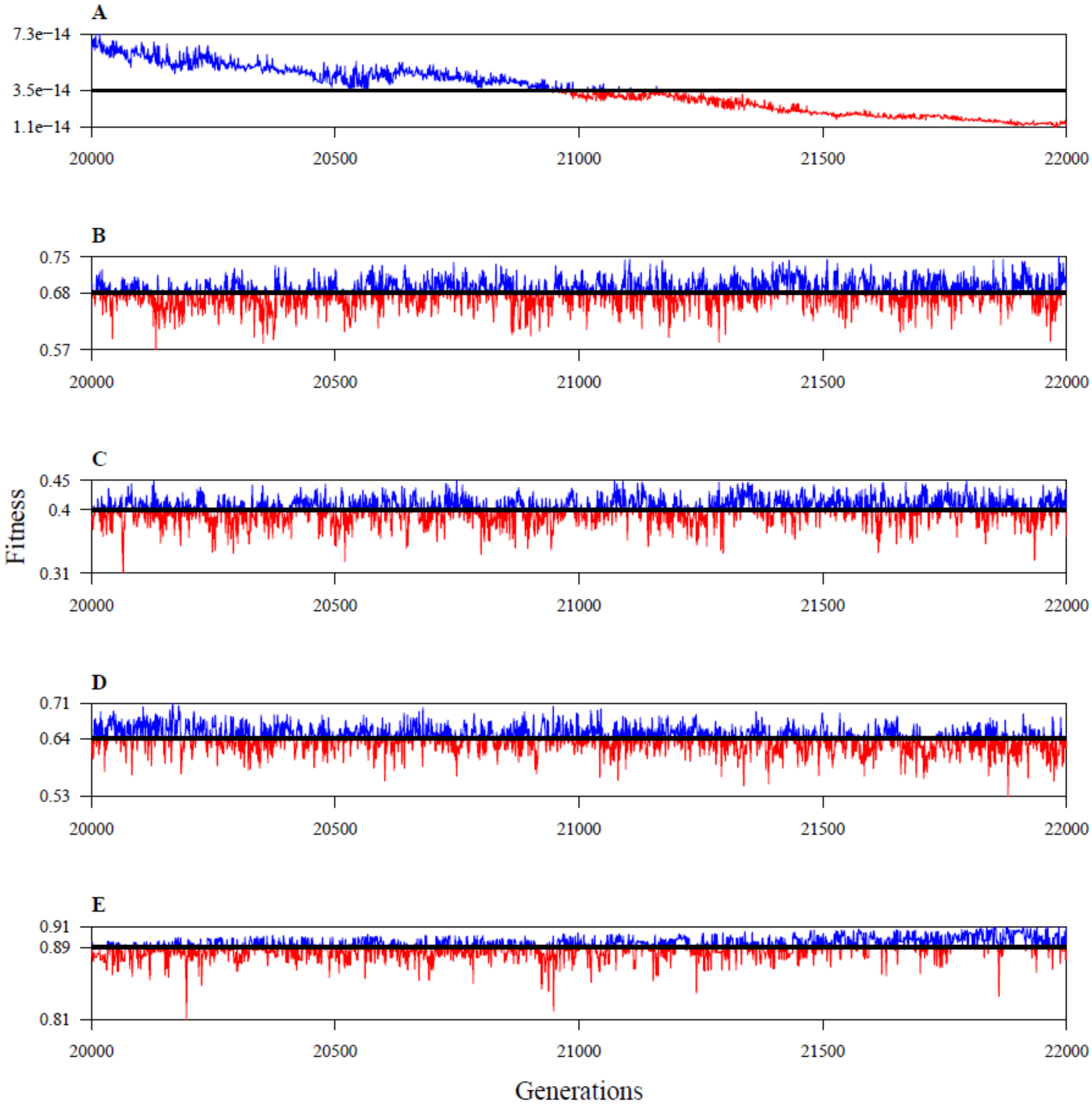

A
7.3e−14
3.5e−14
1.1e−14
20000
20500
21000
21500
22000
B
0.75
0.68
0.57
C
0.45
0.4
0.31
Fitness
D
0.71
0.64
0.53
E
0.91
0.89
0.81
Generations

Figure 22

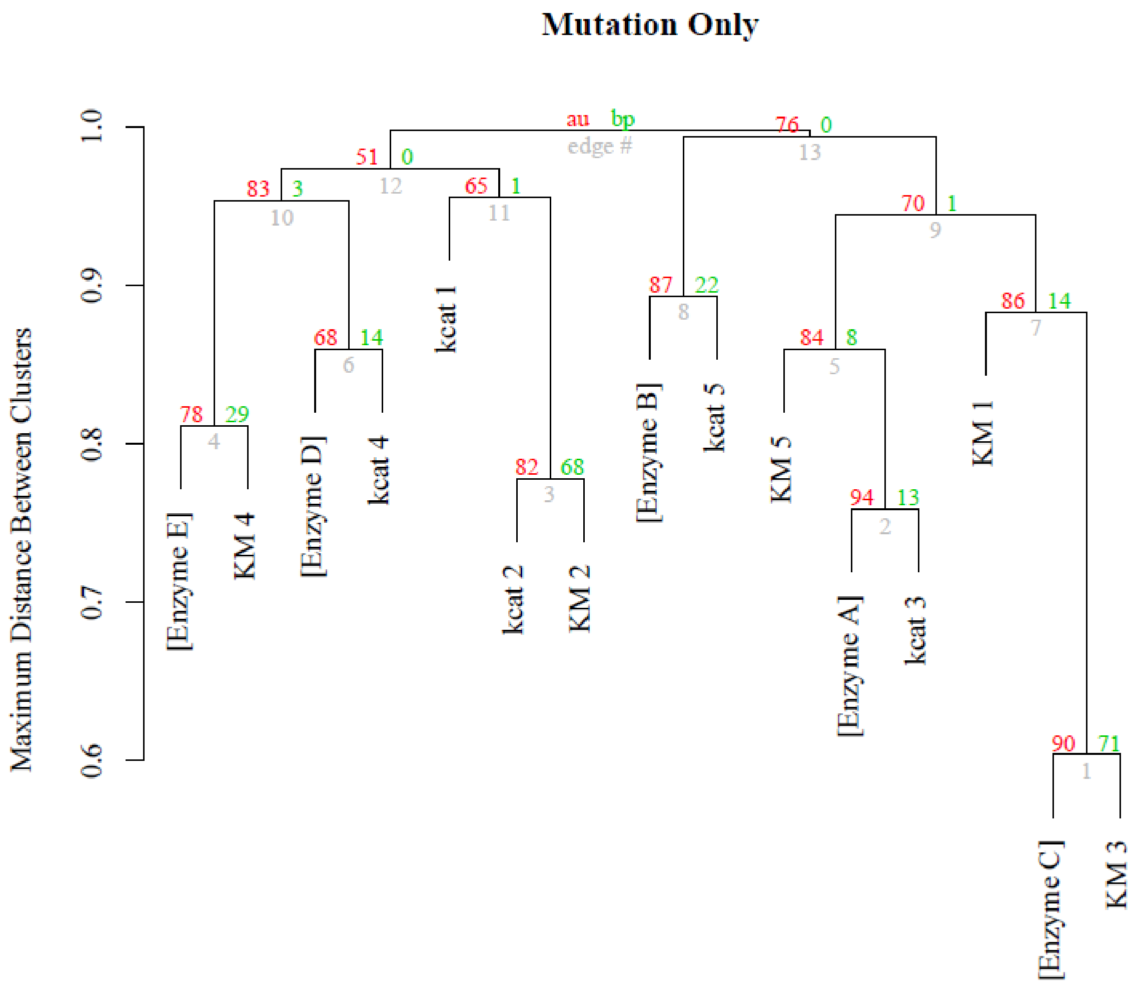
Mutation Only
Maximum Distance Between Clusters
1.0
0.9
0.8
0.7
0.6
au
bp
edge #
[Enzyme E]
KM 4
[Enzyme D]
kcat 4
kcat 1
kcat 2
KM 2
[Enzyme B]
kcat 5
KM 5
[Enzyme A]
kcat 3
KM 1
[Enzyme C]
KM 3
76
0
13
51
0
12
83
3
10
65
1
11
70
1
9
87
22
8
86
14
7
68
14
6
84
8
5
78
29
4
82
68
3
94
13
2
90
71
1

Figure 23

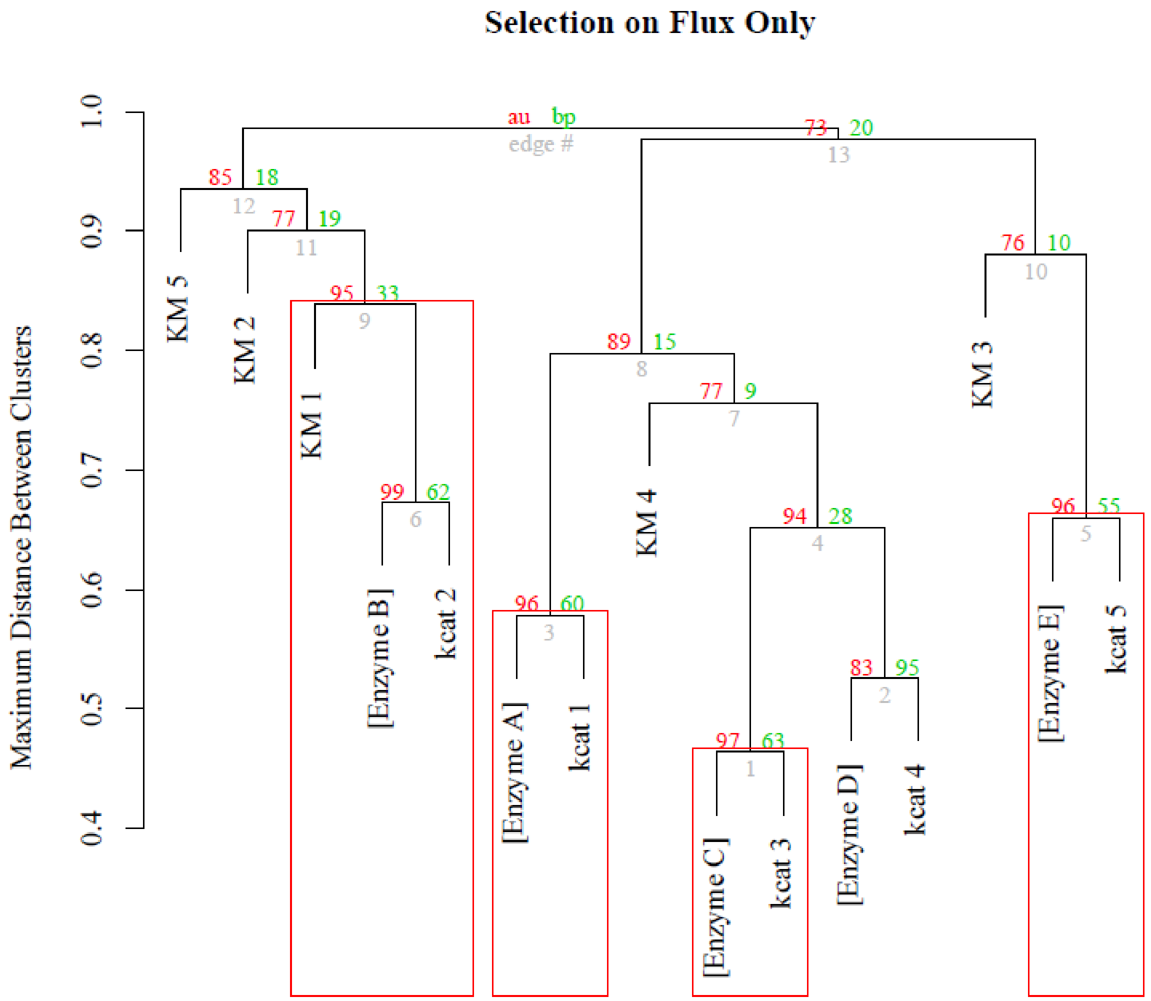
Selection on Flux Only
Maximum Distance Between Clusters
0.4
0.5
0.6
0.7
0.8
0.9
1.0
au
bp
edge #
KM 5
KM 2
KM 1
[Enzyme B]
kcat 2
[Enzyme A]
kcat 1
KM 4
[Enzyme C]
kcat 3
[Enzyme D]
kcat 4
KM 3
[Enzyme E]
kcat 5
85 18 12
77 19 11
95 33 9
99 62 6
73 20 13
89 15 8
96 60 3
77 9 7
94 28 4
97 63 1
83 95 2
76 10 10
96 55 5

Figure 24

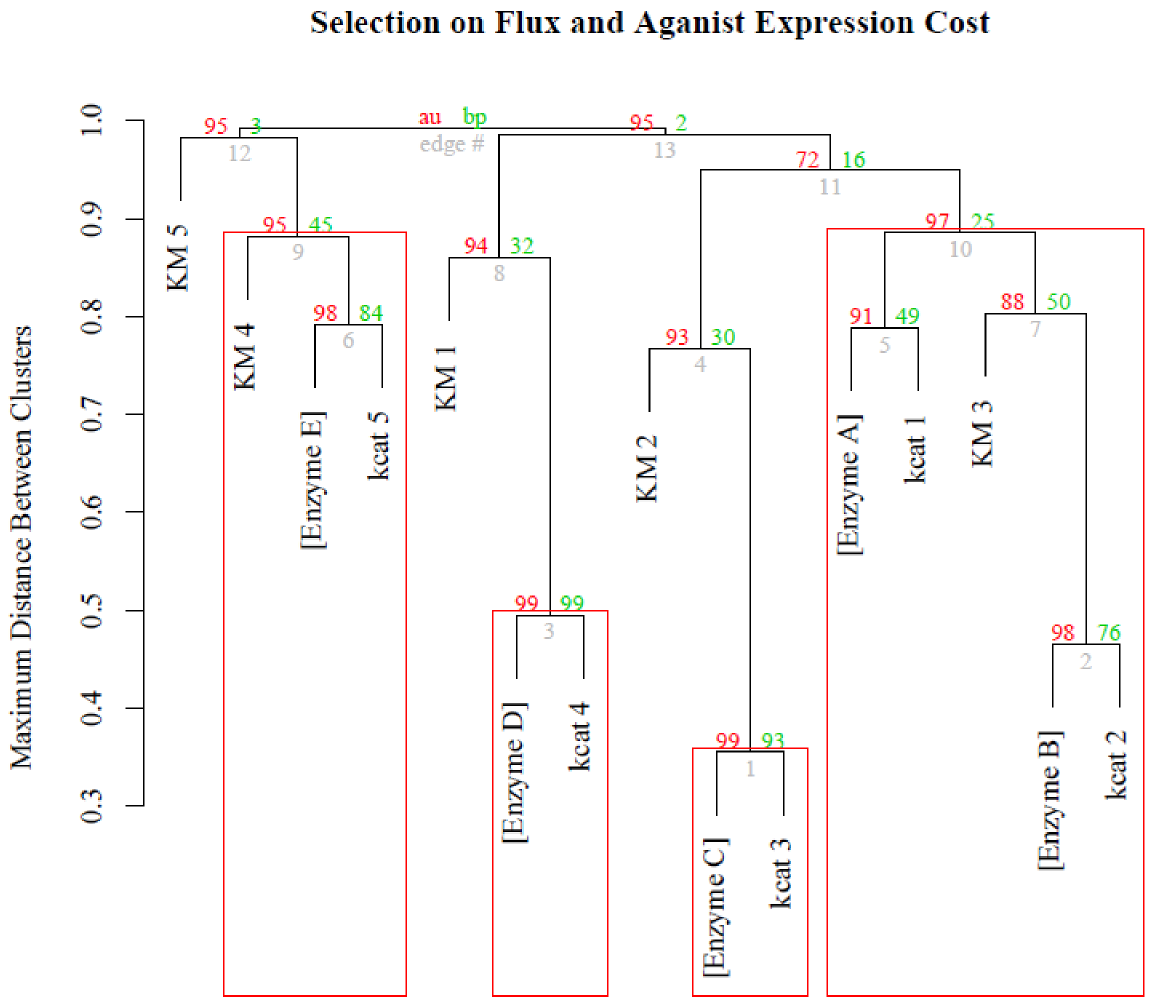
Selection on Flux and Aganist Expression Cost
Maximum Distance Between Clusters
0.3
0.4
0.5
0.6
0.7
0.8
0.9
1.0
au
bp
edge #
KM 5
KM 4
[Enzyme E]
kcat 5
KM 1
[Enzyme D]
kcat 4
KM 2
[Enzyme C]
kcat 3
[Enzyme A]
kcat 1
KM 3
[Enzyme B]
kcat 2

Figure 25

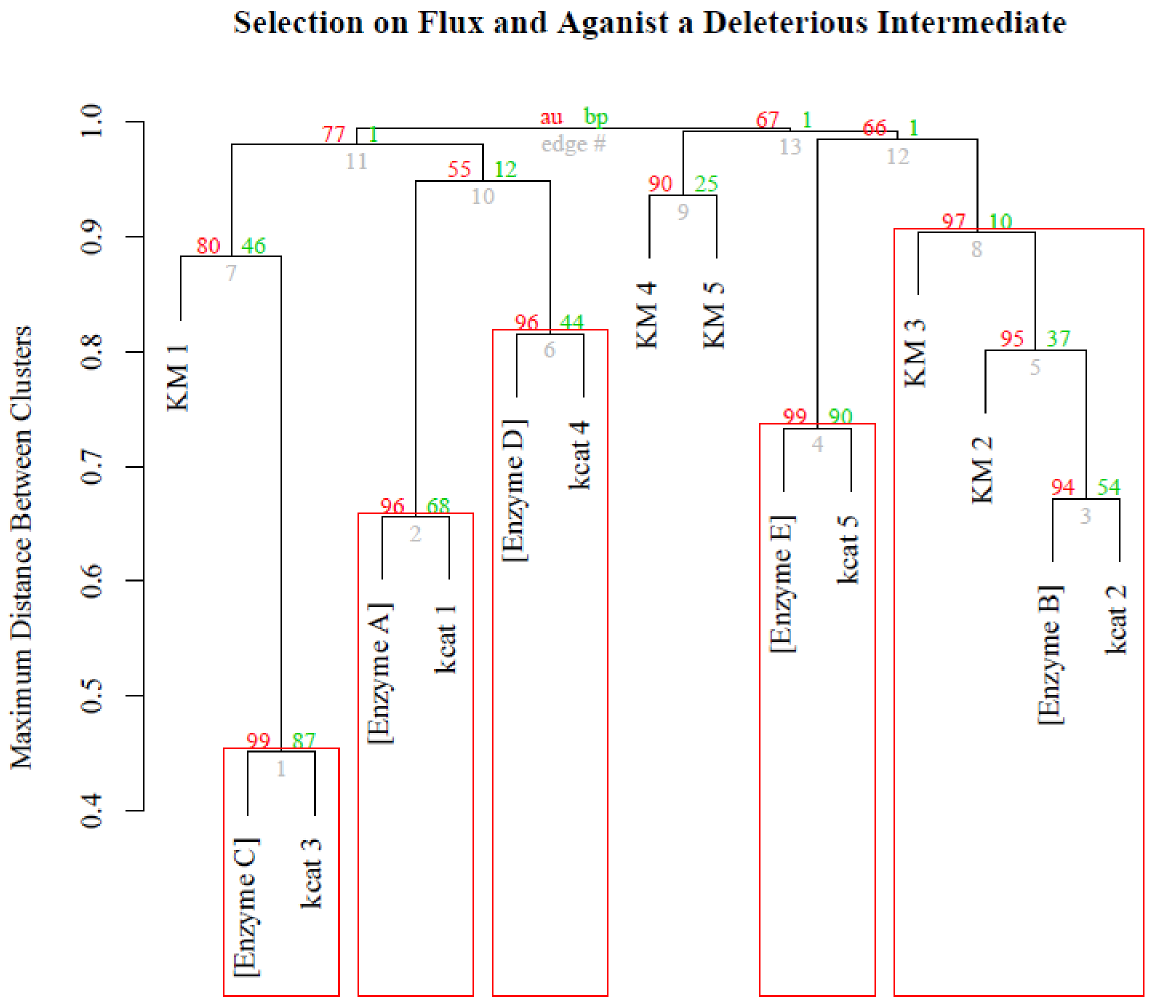

Selection on Flux and Aganist a Deleterious Intermediate
Maximum Distance Between Clusters
0.4
0.5
0.6
0.7
0.8
0.9
1.0
au
bp
edge #
KM 1
[Enzyme C]
kcat 3
[Enzyme A]
kcat 1
[Enzyme D]
kcat 4
KM 4
KM 5
[Enzyme E]
kcat 5
KM 3
KM 2
[Enzyme B]
kcat 2

Figure 26

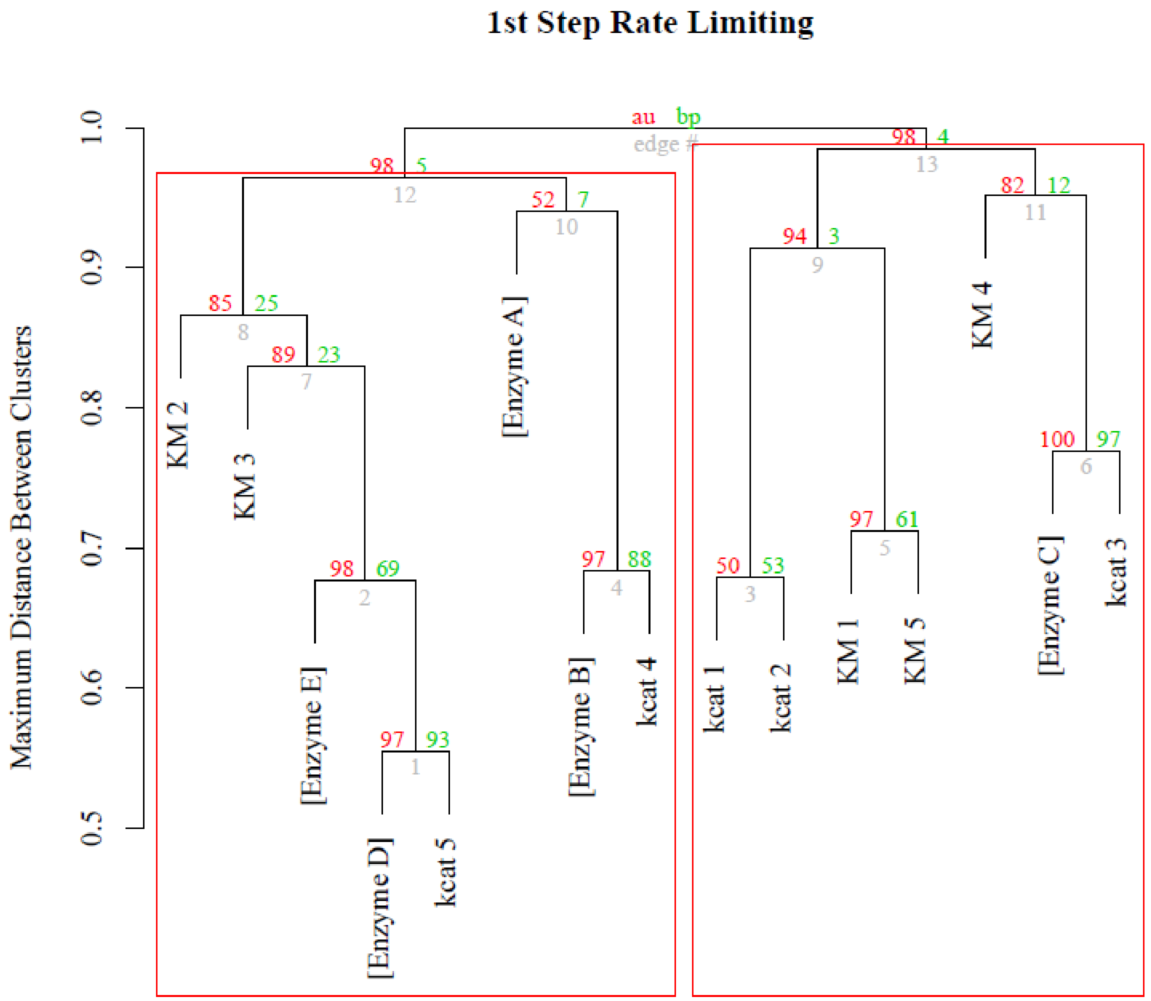
1st Step Rate Limiting
Maximum Distance Between Clusters
0.5
0.6
0.7
0.8
0.9
1.0
au
bp
edge #
KM 2
KM 3
[Enzyme E]
[Enzyme D]
kcat 5
[Enzyme A]
[Enzyme B]
kcat 4
kcat 1
kcat 2
KM 1
KM 5
KM 4
[Enzyme C]
kcat 3
